# Molecular Networking and Bioassay-Guided Preparation and Separation of Active Extract and Constituents from *Vicia tenuifolia* Roth

**DOI:** 10.3390/antiox12101876

**Published:** 2023-10-18

**Authors:** Duc Dat Le, Soojung Yu, Thinhulinh Dang, Mina Lee

**Affiliations:** College of Pharmacy, Research Institute of Life and Pharmaceutical Sciences, Sunchon National University, 255 Jungangno, Suncheon 57922, Jeonnam, Republic of Korea; ddle@scnu.ac.kr (D.D.L.); 1223002@s.scnu.ac.kr (S.Y.); 1220173@s.scnu.ac.kr (T.D.)

**Keywords:** *Vicia tenuifolia*, molecular networking, extraction and purification, anti-inflammatory, molecular docking

## Abstract

Molecular networking drove the selection of material from *V. tenuifolia* organs that targeted active flavonoid glycosides. To optimize the extraction process, the flowers of *V. tenuifolia* were used to produce an anti-inflammatory extract. The effects of variables—organic solvent ratio; extraction time; and temperature—were investigated by the response of anti-inflammatory activity. Bioactivities-guided experiments helped identify fractions with high total phenolic and flavonoid content as well as antioxidant potential. Furthermore, one new compound (**1**), 19 first isolated together, and two known compounds were obtained and identified from the active fraction of this plant. Among them, compounds (**15** and **22**) were first reported for nuclear magnetic resonance (NMR) data from this study. All the isolates were evaluated for their anti-inflammatory capacity throughout, modulating nitric oxide (NO), interleukin (IL)-1β, and IL-8 production. Active compounds were further investigated for their regulation and binding affinity to the inducible nitric oxide synthase (iNOS) and cyclooxygenase-2 (COX-2) proteins by Western blot and in silico approaches, respectively. The findings of this study suggested that the developed extract method, active fraction, and pure components should be further investigated as promising candidates for treating inflammation and oxidation.

## 1. Introduction

Inflammatory bowel disease (IBD) is a term that refers to both ulcerative colitis and Crohn’s disease. Its pathogenesis corresponds to dysregulation of the immune system involving gut flora [1]. Upon activation of the immune system, proteins may induce cytokines and chemokines derived from cells involved in immune responses. An increase in their levels may affect either the synthesis or secretion of reactive oxygen species, prostaglandins, and nitric oxide in inflammatory cascades. These cytokines are beneficial in maintaining the integrity of the intestinal mucosa through their interactions with harmful bacteria [2]. On the other hand, excess cytokine production exacerbates the inflammatory process of IBD [3]. There is no cure for IBD. However, cytokine-based therapies may offer greater specificity to prevent the development of IBD symptoms, thus reducing disease progression and negative effects [4].

Legumes contain high levels of protein, carbohydrates, vitamins, and minerals. They have a low glycemic index, are low in fat, and are cholesterol-free. Thus, the consumption of legumes may have many health benefits due to their high energy content. In particular, legumes may support the consumption of high calories with a low blood glucose level to improve glycemic, cholesterol, and lipid control [5,6]. Among legumes, *Vicia tenuifolia* is a health-promoting food with pharmacological potential due to its content of minerals, proteins, and nutrients such as amino acids, fatty acids, lectins, and albumins [7,8]. Previous reports indicated that the polyphenol content of *V. tenuifolia* may be associated with antioxidant activity [9] and antiproliferative effects [7]. Our previous report [10] revealed the anticancer capacity of *V. tenuifolia*. However, there is no study to investigate the anti-inflammatory effect of fractions and constituents from this plant growth in Korea.

Isolation and purification of chemicals from natural sources consumes much time and effort by researchers, as well as experimental costs, when we re-isolate the known structures of molecules already described. In this study, we performed an effective method using dereplication for preliminary structural assessment to save the time and cost of experiments by evaluating and inferring the structures of molecules predicted in the extract through online databases of natural product compounds [11]. The above method may detect the known compounds from the extracts by separating the molecular constituents, subsequently achieving their spectral signatures, and then searching for or predicting candidates having identical properties from online library data (Global Natural Product Social Molecular Networking, GNPS) [12] or using cheminformatics programs [13,14]. By this way, many novel compounds and their derivatives [12,13,15] were rapidly obtained, with a higher priority for the discovery of natural products.

Continuing our efforts to find active constituents from natural sources [16,17] for the treatment of inflammatory diseases, we found that the total extract of *V. tenuifolia* had a significantly potent anti-inflammatory effect once extraction methods were optimized by using a non-toxic organic product. Thus, this study supported an effective technique for extracting the bioactive components of *V. tenuifolia* for industrial applications. Herein, we establish an extraction method by optimizing variables such as solvent ratio, extraction time, and temperature. In contrast, the assessment of total phenolic and flavonoid contents and antioxidant and anti-inflammatory activities aimed to identify the bioactive fraction and separate the active components. A total of 22 compounds were isolated and identified from this plant. These isolates were also evaluated for their anti-inflammatory activities through both in vitro and in silico studies, as well as Western blotting assays.

## 2. Materials and Methods

### 2.1. Plant Materials

The flowers of *V. tenuifolia* were collected from the herbal garden at Sunchon National University (Suncheon, Korea) in May 2021 and identified by Professor Mina Lee (College of Pharmacy, Sunchon National University). A voucher specimen (SCNUP 32) was stored at the Pharmacognosy Laboratory, College of Pharmacy, Sunchon National University, Suncheon-si, Jeonnam-do, Korea.

### 2.2. Selection of Organs for Extraction

The dried herbs, flowers, and fruits of *V. tenuifolia* were extracted using the same method according to our previous report [17], and then concentrated in vacuo to obtain total extracts, respectively. These extracts were further dissolved in methanol and filtered through polytetrafluoroethylene membrane filters before LC-MS/MS analysis.

### 2.3. LC-MS/MS Conditions and Molecular Network Experiments

Three total extracts were analyzed using our previous method [18] with a slight modification. Particularly, mobile phase elution of a gradient solvent system consisted of channel A (pure water containing 0.1% formic acid) and channel B (acetonitrile, 0.1% formic acid) as follows: 5–15% (B) for 0–4 min, 4–35% (B) for 4–12 min, 35–45% (B) for 12–17 min, 45–100% (B) for 17–23 min and held for three minutes, and 100–5% (B) for 1 min before re-equilibrium with 5% (B).

### 2.4. Selective Optimization Using Organic Solvent, Time, and Temperature for Extraction

The dried flowers (538.4 g) of *V. tenuifolia* were ground into powder and stored in the refrigerator for further experiments. Firstly, this powder (2 g) was extracted with the same volume of 20 mL of different solvent systems, including methanol in distilled water (DW) at 0%, 25%, 50%, 75%, and 100%, and ethanol in DW at 25%, 50%, 75%, and 100%, by sonification for 90 min to obtain nine total extracts, respectively. To optimize the time and temperature extraction, the same amount of dried material was extracted with 100% EtOH during different periods of 30, 60, 90, and 120 min or different temperatures of 25, 30, 40, and 50 °C effort total extracts, respectively.

### 2.5. Antioxidant Assay

The DPPH and ABTS radical scavenging activities were performed by following our previous method [19].

### 2.6. Total Phenolic Content (TPC) Assay

Our previous publication [17] revealed that flavonoid glycosides were active compounds targeting anti-inflammatory activities. Thus, the total phenolic content was determined using a colorimetric method [20] with a slight modification. At first, total extract and gallic acid were dissolved in 1 mL of DMSO to obtain stock solutions. Then, they were diluted 10 times with deionized water. Then, 100 µL of each of the resulting mixtures were mixed with deionized water (1 mL). Folin–Ciocalteu’s phenol reagent (100 µL) was added to each mixture at 0 min. Then, a Na_2_CO_3_ solution (7%, 1 mL) was added at 6 min. At 90 min, the absorbance was measured at 750 nm using a spectrophotometer. The total phenolic content is presented as “mg gallic acid equivalents (GAE)/g extract”. Gallic acid was used as a standard material in ranges of 16.125 to 1000 (μg/mL) to build the calibration curves.

### 2.7. Total Flavonoid Content (TFC) Assay

Colorimetric analysis was assayed by the aluminum chloride colorimetric method [21]. In particular, 2 mL of methanol was added to a 0.5 mL sample (catechin, extract, or fraction). Then, 0.15 mL of NaNO_2_ (1.0 M) was added and vortex mixed. Approximately 3 min later, 0.15 mL of AlCl_3_ (10% *w*/*v*) was added, followed by vortex mixing and 3 min of equilibration time. Then, 1.0 mL of NaOH (1 M) was added. The final volume was adjusted to 5.0 mL by using methanol. All the solutions were vortex mixed after the last step, and the tubes remained in the dark for 40 min before measuring their absorbance at 415 nm by using a spectrophotometer. Catechin was used as a standard in ranges of 6.25 to 1000 (μg/mL) to build the calibration curves.

### 2.8. Extraction and Separation of Compounds (***1***–***22***)

According to the screening results of the NO assay, the optimal extract method was employed for the extraction of dried flowers from *V. tenuifolia*. In detail, this material was extracted with 100% EtOH by sonification for 90 min at 25 °C and then concentrated at vacuo pressure to yield 91.2 g of total extract. Subsequently, this total extract was successfully partitioned with increasing solvent polarity with *n*-hexane (Hex), methylene chloride (MC), and *n*-butanol (Bu) solvents to obtain Hex (19.8 g), MC (0.7 g), Bu (28.8 g) fractions, and water residue (DW, 41.9 g), respectively. These fractions were also evaluated for their inhibition of NO production in LPS-stimulated RAW264.7 cells. Reasonably, the Bu fraction was selected as the target fraction for isolation based on its bioactivities, TPC, and TFC contents. The active Bu fraction was subjected to a Biotage MPLC [eluting with a gradient solvent system of methanol in water (containing 0.1% formic acid) from 30% to 100% for 85 min; flow rate of 20 mL/min; UV detection at 210 and 254 nm; Column Biotage^®^ Sfär C_18_ 120 g, Biotage, Uppsala, Sweden) to obtain eight fractions (Bu1–Bu8). Subfraction Bu2 (1.2 g) was isolated to HPLC using Triart C_18_ column (10 × 250 mm, 5 μm, YMC, Tokyo, Japan), UV detection at wavelength 254 nm, flow rate 3.0 mL/min, eluting with a mobile phase of water (containing 0.1% formic acid, A) and acetonitrile (B) as a gradient solvent system [0 min (2% B)–55 min (18% B)–60 min (20% B)–72 min (100% B)] to obtain compounds **1** (*t*_R_ 22 min), **3** (*t*_R_ 18 min), **4** (*t*_R_ 17 min), **6** (*t*_R_ 13 min), **8** (*t*_R_ 10 min), and **9** (*t*_R_ 8 min). Fraction Bu5 was subjected to a prep HPLC using a Triat C_18_ column (10 × 250 mm, 5 µm), UV detection at wavelength 265 nm, flow rate 3.0 mL/min, eluting with a gradient solvent system from 0 min (19% B) to 85 min (100% B) to obtain compounds **2** (*t*_R_ 38 min), **10** (*t*_R_ 17 min), **15** (*t*_R_ 27 min), and further purified using the same conditions by exchange the isocratic elution of 17% B for 65 min, compound **13** (*t*_R_ 49 min) and **20** (*t*_R_ 52 min). Fraction Bu6 was subjected to a prep HPLC using a Triat C_18_ column (10 × 250 mm, 5 µm), UV detection at wavelength 265 nm, flow rate 3.0 mL/min, eluting with a gradient solvent system from 0 min (19% B) to 85 min (100% B) to obtain compounds **5** (*t*_R_ 14 min), **7** (*t*_R_ 12 min), **11** (*t*_R_ 69 min), **12** (*t*_R_ 43min), **14** (*t*_R_ 53min), **17** (*t*_R_ 37min), **18** (*t*_R_ 56min), **19** (*t*_R_ 61min), and further purified using the same conditions by exchanging the isocratic elution of 17% B for 80 min effort compounds **16** (*t*_R_ 43min), **21** (*t*_R_ 65min), and **22** (*t*_R_ 50 min).

#### Spectroscopic Data of Compounds **1**, **15**, and **22**

Vicia D (**1**): Yellowish syrup; ${\left[\alpha \right]}_{D}^{26}$ − 35.6 (c 0.025, MeOH); UV (MeOH) *λ*_max_ (log ε) 202 (3.95), 221 (3.85), 266 (3.80) nm; ^1^H NMR (400 MHz, DMSO-d_6_) and ^13^C NMR (100 MHz, DMSO-d_6_) data see Table 1; HR-ESI-MS m/z 657.1632 [M-H]^−^ (calc. for C_29_H_37_O_16_, 657.1667), 511.1080 [M-H-Rha]^−^, 311.0755 [M-3H-Rha-Glc-2OH]^−^, 162.0394 [Glc]^−^, 147.2635 [Rha]^−^.

Approximately 6-Methoxykaempferol 3-O-sophoroside (**15**): Yellowish syrup; ${\left[\alpha \right]}_{D}^{26}$+15.8 (c 0.05, MeOH); UV (MeOH) *λ*_max_ (log ε) 214 (3.75), 253 (1.50), 348 (4.10) nm; ^1^H NMR (400 MHz, DMSO-d_6_) and ^13^C NMR (100 MHz, DMSO-d_6_) data see Table 1; HR-ESI-MS m/z 641.1710 [M+H]^+^ (calc. for C_28_H_32_O_17_H, 641.1718), 317.1349 [M+H-Glc-Glc]^+^, 179.0633 [Glc]^+^.

Diosmetin 7-O-(2″-apiosyl)-glucoside (**22**): Yellowish syrup; ${\left[\alpha \right]}_{D}^{26}$ − 55.6 (c, MeOH); UV (MeOH) *λ*_max_ (log ε) 214 (4.05), 254 (4.00), 344 (3.95) nm; ^1^H NMR (400 MHz, DMSO-d_6_) and ^13^C NMR (100 MHz, DMSO-d_6_) data see Table 1; HR-ESI-MS m/z 595.1650 [M+H]^+^ (calc. for C_27_H_30_O_15_H, 595.1663), 301.1406 [M+H-Glc-Api]^+^, 179.0633 [Glc]^+^, 149.0231 [Api]^+^.

### 2.9. Anti-Inflammatory Assay

#### 2.9.1. Cell Culture and Cell Viability

Cell culture and viability were discussed in our previous report [22]. Briefly, human colon epithelial (HT-29) and mouse macrophage (RAW264.7) cells were maintained in Dulbecco’s modified Eagle’s medium (DMEM), followed by adding 10% FBS, streptomycin sulfate (100 µg/mL), and penicillin (100 IU/mL). Then, they were incubated in a humidified atmosphere of 5% CO_2_ at 37 °C. Then, they were seeded into 96-well plates. After incubation for 24 h, they were treated with samples (total extract, fractions, or isolates) and stimulated with LPS, respectively. The cell viability was assessed using an MTT assay. The absorbance of the formazan crystals was carried out at 570 nm in a microplate reader (Bio Tek Instruments, Winooski, VT, USA).

#### 2.9.2. Measurement of NO Production

No assay was followed in our previous study [16]. The level of NO production was determined by measuring the amount of secreted nitrite from the cell culture supernatants. In detail, RAW264.7 cells (1 × 10^5^ cells/well) were pretreated with samples (total extract, fractions, 100 μg/mL, and isolates **1**–**22**, 100 μM). After 1 h, RAW264.7 cells were stimulated with LPS (1 μg/mL) and continuously incubated for 16 h. Then, the collected medium was supplemented by the same volume of Griess reagent (*w*/*v*, 1%), sulfanilamide (*v*/*v*, 5%), phosphoric acid (*w*/*v*, 0.1%), and N-(1-naphtyl) ethylenediamine at room temperature. After 10 min, the absorption was determined at 550 nm. The NO production was calculated from treated samples and controls.

#### 2.9.3. Measurement of IL-8 Production

The IL-8 production assay [19] was used on HT-29 cells (3 × 10^5^ cells/well) in 96-well plates using an ELISA kit (BD OptEIATM, San Jose, CA, USA) following the manufacturer’s instructions.

#### 2.9.4. IL-1β Assay

The assay was to evaluate the expression of IL-1β in RAW264.7 cells under LPS stimulation. RAW264.7 cells were incubated with compounds (**1**–**22**, 100 μM) for 2 h. Then, RAW264.7 cells were stimulated with LPS (1 μg/mL) and continuously incubated for 20 h. The level of IL-1β in the culture medium was measured by an ELISA kit (Invitrogen, cat. no. KHC0011; Thermo Fisher Scientific, Inc., Waltham, MA, USA) following the manufacturer’s instructions.

### 2.10. In Silico Study

Molecular docking studies were performed using MGL 1.5.6 tools. The structures of proteins [iNOS (PDB ID: 3E7G), COX-2 (PDB ID: 5IKQ), and IL-8 (PDB ID: 5D14)] were obtained from the RCSB protein data bank (https://www.rcsb.org; accessed on 5 June 2023). They were cleaned, and they added hydrogen atoms as well as Kollman charges. The structure of Vicia D (**1**) was prepared and minimized by the Avogadro package via the force field method (MMFF94). Other structures (**8**, **11**, **13**, **15**, **16**, **20**, and **22**) were downloaded from PubChem (https://pubchem.ncbi.nlm.nih.gov (accessed on 6 June 2023)). Then, these obtained data were converted into the pdbqt file format. The ligand optimization was assessed by adding Gasteiger charges. Then, the Lamarckian genetic algorithm with default parameters was applied for a total of 100 runs. The binding affinity calculations were performed using AutoDock 4.2 [17]. The visualization was expressed using the Pymol and Discovery Studio 2021 tool programs.

### 2.11. Western Blotting Assay

The reduction levels of iNOS and COX-2 protein expression on RAW264.7 cells by the strong active compounds were tested following our previous study [17].

### 2.12. Statistical Analysis

Data were expressed as the mean ± SD (*n* = 3) from at least three independent experiments. Graphprism version 8.0.1 software (Graphpad Software, La Jolla, CA, USA) was applied for statistical analysis. The obtained values were evaluated by one-way ANOVA analysis, followed by Tukey’s multiple comparison test. Differences were considered to be significant at * *p* < 0.05 and ** *p* < 0.01, compared to controls.

## 3. Results

### 3.1. Selection of V. tenuifolia Organ Based on Molecular Networking Guidance

**Figure 1:**
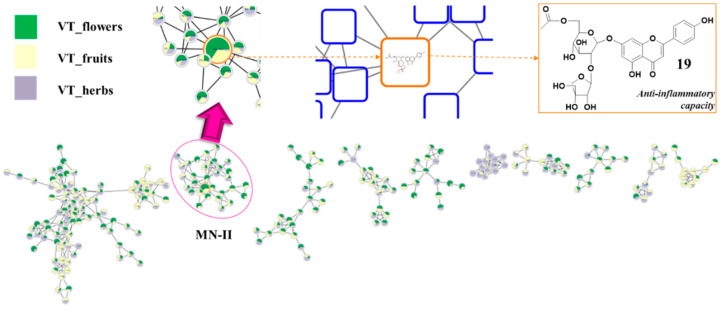
Molecular networking analysis of total extracts of different organs [flower (green), fruits (yellow), and herbs (orchid)] of *V. tenuifolia*. A detailed view of the MN-II cluster (round frame) with a node (tangerine frame) of *m/z* 606.1585 pointed to compound potential anti-inflammatory activity by in silico prediction.

Our previous study [11] revealed that 6″-acetylapiin was extracted from other *Vicia* species, showing significant anti-inflammatory capacity. Thus, we targeted flavonoid glycoside derivatives to find the active components in different organs of *V. tenuifolia*. Total extracts of various parts (flowers, fruits, and herbs) of the plant were analyzed by using an untargeted LC-MS/MS workflow process of molecular networking. The dereplication of LC-MS/MS spectral data were conducted using feature-based molecular networking (FBMN) [12], allowing the detection and relative quantification of LC-MS/MS spectral ions via chromatographic extracts. As shown in Figure 1, MN-II showed the presence of the above compound as a highlighted node. The pie chart suggested that this flavonoid glycoside contained a relative abundance from flower extracts higher than that of herbal and fruit extracts. Therefore, the flowers were selected as material for further studies.

### 3.2. Optimization of Extraction Method

**Figure 2:**
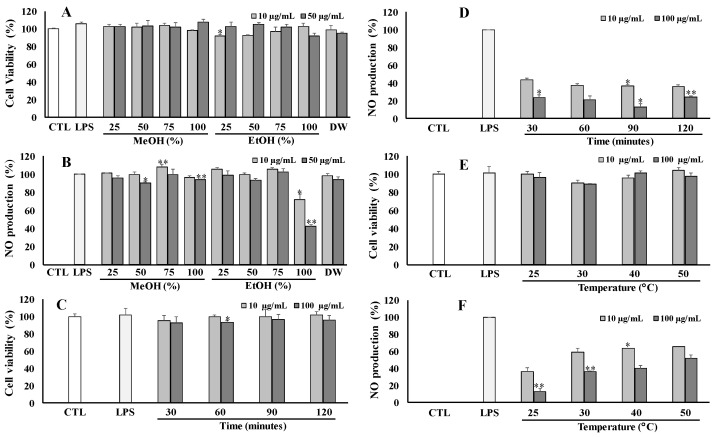
Cytotoxic and NO production of different total extracts of *V. tenuifolia* in LPS stimulated RAW264.7 cells by extracting with different solvent ratios (**A**,**B**), extraction times (**C**,**D**), and temperatures (**E**,**F**), respectively. Each experiment was performed in triplicate. The data are represented as the mean ± SD. * *p* < 0.05, ** *p* < 0.01 vs. LPS-treated group.

The dried flowers of *V. tenuifolia* were extracted with different solvent systems of organic in water, including 25%, 50%, 75%, and 100% (MeOH); 25%, 50%, 75%, and 100% (EtOH); and 100% water effort total extracts, respectively. These extracts were then screened for their anti-inflammatory capacity by modulating NO production in LPS-stimulated RAW264.7 cells at different concentrations of 10 and 50 μg/mL. As shown in Figure 2, the total extract of 100% EtOH exhibited a potential anti-inflammatory effect (inhibition rate of 42.67%) without a cytotoxic effect on RAW264.7 cells at a tested concentration of 50 μg/mL (Figure 2A,B). Thus, the optimal solvent of 100% EtOH was selected for further experiments.

To optimize the extraction time, the same amount of dried materials was extracted for different periods of 30, 60, 90, and 120 min at room temperature (25 °C) by using 100% EtOH. As a result, the residue at 90 min showed the strongest inhibition against NO production at 100 μg/mL (Figure 2D). Thus, an extraction time of 90 min was set up for further experiments. To investigate the temperature effect, the same dried plant was extracted at different temperatures, including room temperature (25 °C), 30 °C, 40 °C, and 50 °C, for 90 min, using a solvent system of 100% EtOH. The obtained result exhibited that the residue at room temperature (25 °C) exhibited the best inhibitory effect against NO production. Whereas, all the tested samples did not cause any effect on the viability of RAW264.7 cells (Figure 2E,F). Finally, the optimal extract conditions were established by extraction at room temperature (25 °C) for 90 min using 100% EtOH organic solvent.

### 3.3. Anti-Inflammatory Capacity of Active Fraction

The dried material was extracted with 100% EtOH at room temperature for 90 min to obtain the total extract. This concentrated extract was then successfully partitioned using increasing polarity solvents to obtain Hex, MC, Bu, and DW fractions, respectively.

**Figure 3:**
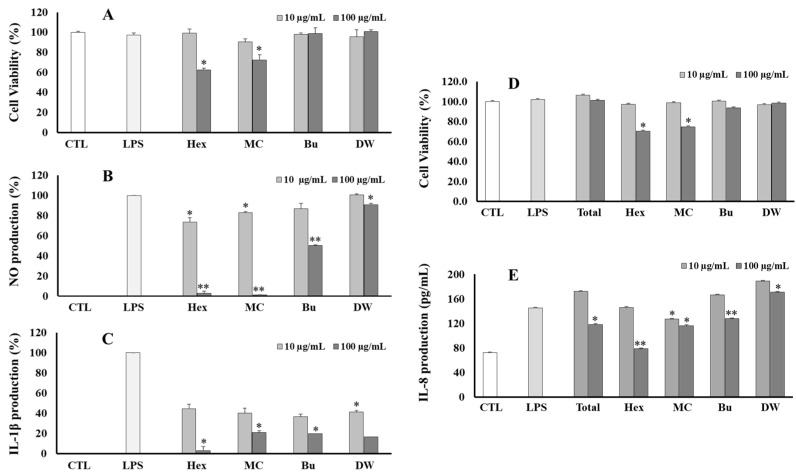
Cytotoxic (**A**,**D**), NO (**B**), IL-1β (**C**), and IL-8 production (**E**) inhibitory effects of total extract or fractions of *V. tenuifolia* on RAW264.7 and HT-29 cells, respectively. Each experiment was performed in triplicate. The data are represented as the mean ± SD. * *p* < 0.05, ** *p* < 0.01 vs. LPS-treated group.

These fractions were also evaluated for anti-inflammatory effects by their inhibition of NO, IL-1β, and IL-8 production in LPS-stimulated RAW264.7 and HT-29 cell lines, respectively. In the NO assay, Hex, MC, and Bu fractions displayed good anti-inflammatory effects against NO production (Figure 3B). In the IL-1β assay, all fractions showed a significant reduction in IL-1β production induced by LPS in RAW264.7 cells (Figure 3C). However, both Hex and MC fractions showed some toxic effects on the viability of RAW264.7 cells. A similar result was also observed in the IL-8 assay (Figure 3D,E). Furthermore, the total extract and fractions were also screened for their inhibition against IL-6 and TNF-α production in LPS-stimulated RAW264.7 cells. However, these samples did not exhibit a significant effect.

### 3.4. Antioxidant Effect, Total Phenolic (TPC), and Total Flavonoids (TFC) Contents of Extract and Fractions

**Figure 4:**
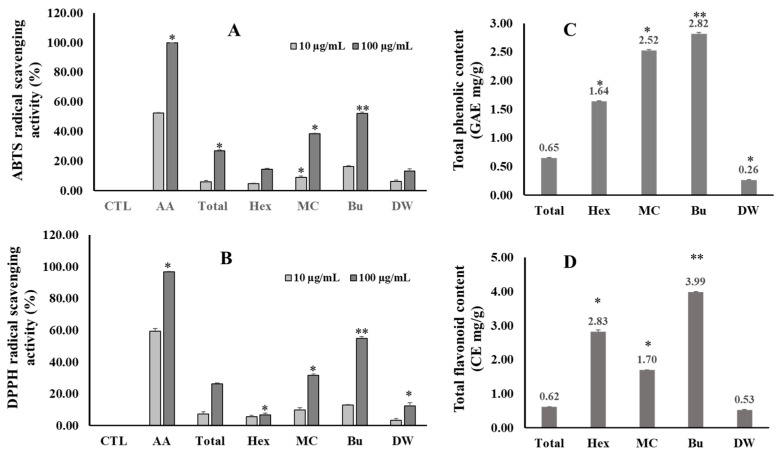
ABTS (**A**) and DPPH (**B**) radical scavenging activity, and the total phenolic (**C**) and flavonoid (**D**) content of the total extract and fractions of *V. tenuifolia*. Each experiment was performed in triplicate. The data are represented as the mean ± SD. * *p* < 0.05, ** *p* < 0.01 vs. control.

The total phenolic content varies among the fractions from 0.26 to 2.82 GAE mg/g total extract. In contrast, the Bu (2.82 ± 0.02 GAE mg/g) fraction showed the highest TPC, followed by MC (2.52 ± 0.02 GAE mg/g), Hex (1.64 ± 0.01 GAE mg/g), and the lowest DW (0.26 ± 0.01 GAE mg/g) fractions (Figure 4). In the TFC assay, the Bu fraction also contained the highest TFC value of 3.99 ± 0.00 CE mg/g, followed by the Hex, MC, and DW fractions. In addition, the total extract and fractions from *V. tenuifolia* also exhibited antioxidant capacity through their scavenging activity against ABTS and DPPH radicals. Among them, the Bu fraction displayed the strongest scavenging activity for both radicals at a concentration of 100 μg/mL. 

After consideration of all the above results, the Bu fraction was selected for separation to find the active constituents.

### 3.5. Structural Elucidation of 22 Constituents

**Figure 5:**
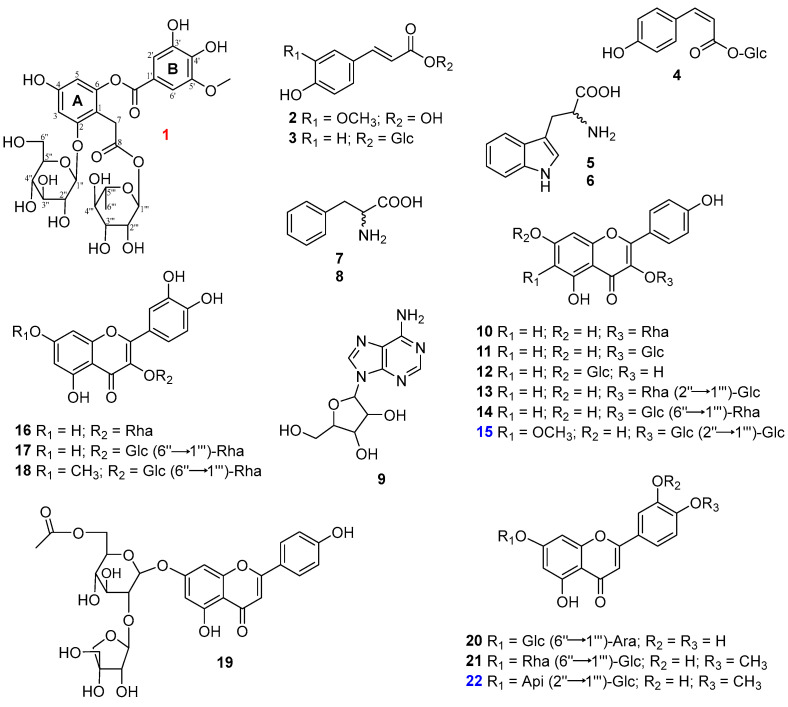
Chemical structures of isolated compounds (**1**–**22**) from *V. tenuifolia.*

**Figure 6:**
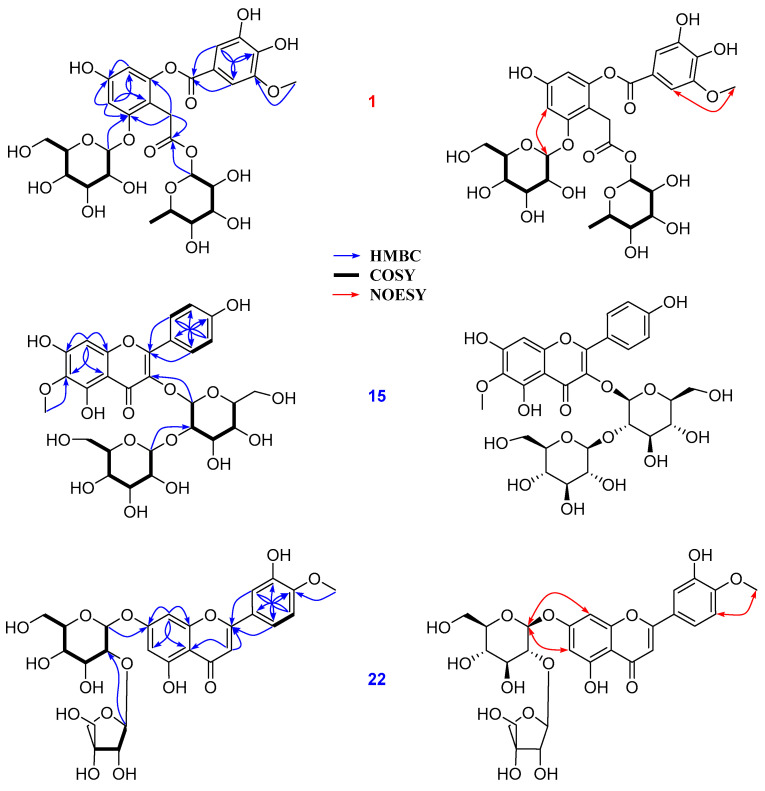
Key COSY, HMBC, and NOESY correlations of compounds **1**, **15**, and **22**.

The separation of the ethanolic total extract resulted in the isolation of 22 compounds, as depicted in Figure 5. Compound **1** showed a primary deprotonated peak at *m*/*z* 657.1632 (calc. for C_29_H_36_O_16_, 657.1667). The mass fragmentation pathway of **1** produced the ion peaks at [M-H]^−^ at *m*/*z* 511.1060 and *m*/*z* 311.0755, which correspond to a reduction of [M-H-rham]^−^ and [M-3H-rham-glc-2OH]^−^, respectively. On the other hand, the MS^2^ ion products showed peaks at *m*/*z* 147.2835 and 162.8927 (Figure S1, Supplementary Materials), revealing the presence of rhamnosyl and glucosyl moieties [23]. In addition, the chemical shift values of glycosidic signals were obtained from their spectroscopic data and compared to those of identical signals from reported values [24,25], respectively. The ^1^H NMR spectrum of **1** revealed the presence of 2,4,6 trihydroxy-aromatic units at *δ*_H_ 6.50 (1H, d, *J* = 2.3 Hz, H-3), 6.21 (1H, d, *J* = 2.3 Hz, H-5), 3,4-dihydroxy-5-methoxybenzoyl [*δ*_H_ 7.16 (1H, d, *J* = 1.9 Hz, H-2′), 7.11 (1H, d, *J* = 1.9 Hz, H-6′), 3.78 (3H, 5-OCH_3_)] units, two anomeric protons at *δ*_H_ 5.65 (1H, d, *J* = 1.8 Hz, H-1‴) and *δ*_H_ 4.66 (1H, d, *J* = 7.3 Hz, H-1″), which are suggested to respectively correspond to *α*-and *β*-glycosidic units, along with a geminal coupling constant in methylene group at *δ*_H_ 3.45 (1H, d, *J* = 17.0 Hz, H_2_-7) and 3.62 (1H, d, *J* = 17.0 Hz, H_2_-7), and a secondary methyl at *δ*_H_ 0.96 (3H, d, *J* = 6.2 Hz, H_3_-6‴) together with other carbonic signals (Table 1). The ^13^C NMR spectrum of **1** revealed 27 signals, including two carboxylic at *δ*_C_ 169.3 (C-8) and 163.8 (C-7’), as well as 12 aromatic and two glycosidic groups that were classified by Dept and HMQC spectra. The spectroscopic data (Figures S1–S8, Supplementary Materials), for **1** consisted of those reported compounds [25] with some differences from the functional group of the second phenyl (B) ring and sugar unit attachment. Briefly, the HMBC spectrum showed the correlations of H-2′ (*δ*_H_ 7.11) to C-4′ (*δ*_C_ 140.3)/C-6′ (*δ*_C_ 111.1)/C-7′ (*δ*_C_ 163.8) and those of H-6′ (*δ*_H_ 7.16) to C-2′ (*δ*_C_ 105.2)/C-4′ (*δ*_C_ 140.3)/C-7′ (*δ*_C_ 163.8), establishing the B-ring partial structure. The HMBC cross-peaks of H-1″ (*δ*_H_ 4.66)/H-3 (*δ*_H_ 6.50) to C-2 (*δ*_C_ 157.0) suggested the Glc-linkage at the C-2 position of the A-ring (Figure 6). Moreover, the HMBC correlations of H-7 (*δ*_H_ 3.45/3.62)/H-1‴ (*δ*_H_ 5.65) to C-8 (*δ*_C_ 169.3) were confirmed to correspond to the rhamnosyl unit linked to the C-8 position (Figure 6). Furthermore, the 5-OCH_3_ and C-2-*O*-glc attachments were supported by NOESY correlations between H-6′ (*δ*_H_ 7.16) and H_3_ (*δ*_H_ 3.78), and those between H-3 (*δ*_H_ 6.50) and H-1″ (*δ*_H_ 4.66) (Figure 6). With the above information, the structure of compound **1** was successfully established and named Vicia D.

Compound **15** displayed characteristic signals corresponding to flavonoid glycosides. In detail, its ^1^H NMR spectrum showed two magnetically equivalent spin systems at *δ*_H_ 8.09 (2H, d, *J* = 8.9 Hz, H-2′/6′) and 6.90 (2H, d, *J* = 8.9 Hz, H-3′/5′), an aromatic proton at *δ*_H_ 6.24 (s, H-8), a methoxy group at *δ*_H_ 3.87 (6-OCH_3_), and two anomeric protons of *β*-glycosidic units at *δ*_H_ 5.45 (1H, d, J = 7.6 Hz, H-1″), 4.74 (1H, d, *J* = 7.4 Hz, H-1‴). ^13^C NMR spectrum of **15** expressed 28 carbons, including 16 signals belonging to aglycon 6-methoxykaempferol and 12 carbonic signals (Table 1). An HMBC cross-peak of H-1‴ (*δ*_H_ 4.77) to C-2″ (*δ*_C_ 82.6) confirmed the second glucose linkage to the C-2″ of the first glucose. This observation explained the downfield shift of C-2″ (*δ*_C_ 82.6). In addition, the HMBC correlation of H-1″ (*δ*_H_ 5.48) to C-3 (*δ*_C_ 134.9) revealed that two glucoses connected to 6-methoxykaempferol aglycone [26] through the C-3 position (Table 1). Signals of the glycosidic units were also assigned and identified by analysis of their chemical shift values, signal correlations from NMR spectra (Figures S10–S15, Supplementary Materials), and comparison to those reported in the literature [27], respectively. Further assignments and correlations of **15** were depicted in Figure 6. With the above evidence, the structure of **15** was established as 6-methoxykaempferol 3-*O*-sophoroside [28].

Compound **22** displayed an ABX spin system [*δ*_H_ 7.57 (1H, dd, *J* = 2.4, 8.6 Hz, H-6′), 7.46 (1H, d, *J* = 2.4 Hz, H-2′), 7.10 (1H, d, *J* = 8.6 Hz, H-5′)]; three olefinic protons at *δ*_H_ 6.84 (1H, s, H-3), 6.79 (1H, s, H-8), 6.44 (1H, s, H-6); a methoxy signal at *δ*_H_ 3.87 (4′-OCH_3_). Two anomeric protons were also observed at *δ*_H_ 5.35 (1H, d, *J* = 1.5 Hz, H-1‴) and 5.18 (1H, d, *J* = 7.3 Hz, H-1″), which respectively correspond to *α*- and *β*-glycosidic units. The ^13^C NMR spectrum of **22** displayed 27 carbons, including 16 signals characterized for a diosmetin backbone [29] and 11 signals assigned for two sugar units. The HMBC correlations of protons at *δ*_H_ 6.79 (1H, s, H-8)/6.44 (1H, s, H-6)/5.18 (1H, d, *J* = 7.3 Hz, H-1″) to C-7 (*δ*_C_ 163.0) indicated C-7-*O*-glc linkage. In addition, HMBC cross-peaks of H-1‴ (*δ*_H_ 5.35) to C-2″ (*δ*_C_ 76.5) together with the COSY correlation between H-1″ (*δ*_H_ 5.18) and H-2″ (*δ*_H_ 3.53) confirmed the Glc-2″-*O*-Api attachment. On the other hand, the HMBC cross-peak of H_3_-4′ (*δ*_H_ 3.87) to C-4′ (*δ*_C_ 151.3) as well as the NOESY correlation between a singlet methoxy at *δ*_H_ 3.87 and a doublet at *δ*_H_ 7.10 (1H, d, *J* = 8.6 Hz, H-5′) confirmed the 4′-OCH_3_ connection. Cross-peaks of H-1″ (*δ*_H_ 5.18) to H-6 (*δ*_H_ 6.44)/H-8 (*δ*_H_ 6.79) were observed in the NOESY spectrum (Figure 6). This information confirmed the 7-glc attachment. The observation of chemical shift values of glycosidic signals agreed with those reported in the literature [30]. Further identification of mass fragments and spectroscopic data were provided (Figures S16–S22, Supplementary Materials), Based on the above evidence, the structure of **22** was established as diosmetin 7-*O*-(2′’-apiosyl)-glucoside.

There were also other compounds that were isolated and identified as ferulic acid (**2**) [31], *trans*-*p*-coumaroyl-D-glucopyranose (**3**) [32], *cis*-*p*-coumaroyl-D-glucopyranose (**4**) [33], L-tryptophan (**5**), and D-tryptophan (**6**) [34], D-phenylalanine (**7**), L-phenylalanine (**8**), and adenosine (**9**) [35], Afzelin (**10**) [36], rhamnocitrin 3-*O*-glucoside (**11**) [37], kaempferol 7-*O*-glucoside (**12**) [38], kaempferol 3-*O*-(2″-*O*-*β*-D-glucopyranosyl)-*α*-L-rhamnopyranoside (**13**) [39], kaempferol 3-*O*-rutinoside (**14**) [40], quercetin-3-*O*-rhamnoside (**16**) [41], rutin (**17**) [42], rhamnetin 3-*O*-rutinoside (**18**) [43], 6″-acetylapiin (**19**) [11], graveobioside A (**20**) [44], and diosmetin 7-*O*-rutinoside (**21**) [45] by analysis of their spectroscopic data compared to those reported from literature (Figure 4).

### 3.6. Biological Activities of Isolated Compounds

**Figure 7:**
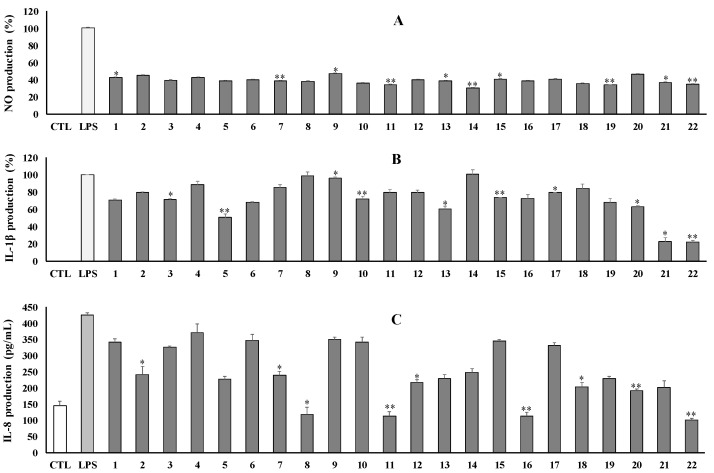
Inhibitory effects on NO (**A**), IL-1β (**B**), and IL-8 (**C**) production of isolated compounds (**1**–**22**) at 100 μM. Each experiment was performed in triplicate. * *p* < 0.05, ** *p* < 0.01.

The isolated compounds (**1**–**22**) were evaluated for their inhibition of LPS-induced NO production in RAW264.7 cells. Most compounds reduced NO production at a tested concentration of 100 μM, with inhibition rates ranging from 55% to 69% (Figure 7A). Meanwhile, compounds (**9** and **20**) showed weak effects with inhibition rates of 53.0% and 53.5%, respectively. By contrast, no isolates show a significant cytotoxic effect on RAW264.7 cells (Figure S23, Supplementary Materials).

Moreover, all isolates were also evaluated for their inhibition against IL-1β production induced by LPS on the RAW264.7 cell line. As a result, compounds **21** and **22** strongly inhibited IL-1β production at tested conditions by approximately 77% inhibition rates. Compounds **5** and **13** also exhibited moderate inhibition. Meanwhile, other compounds showed a weak to inactive effect against IL-1β production. No isolates showed any significant effect on the viability of RAW264.7 cells (Figure 7B).

The above compounds were also examined for their anti-inflammatory effect against IL-8 production in LPS-stimulated HT-29 cells. As shown in the figure, compounds (**8**, **11**, **16**, and **22**) strongly inhibited IL-8 production at concentrations of 100 μM. Other compounds showed moderate to weak inhibition at the same tested conditions (Figure 7C). No compounds caused any effect on the viability of HT-29 cells (Figure S23, Supplementary Materials).

### 3.7. Molecular Docking Analysis

**Figure 8:**
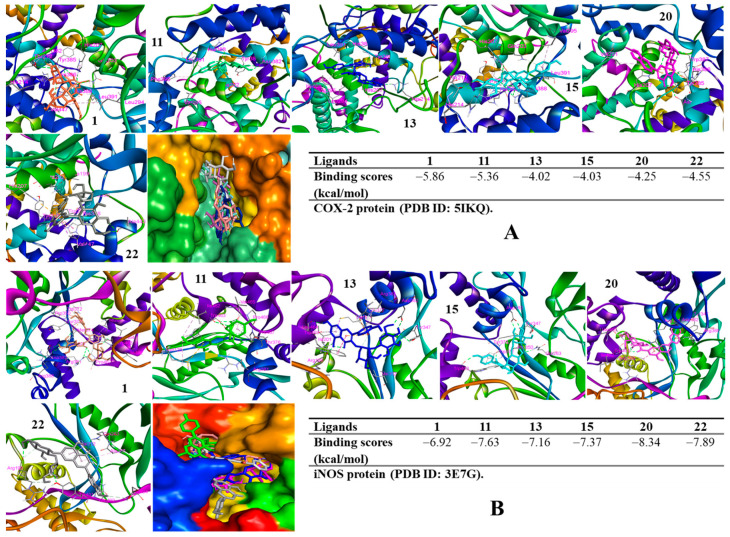
Visualization and interacting residues in the 5IKQ-compound complexes (**A**) and 3E7G-compound complexes (**B**).

To verify the anti-inflammatory properties of active constituents, in silico approaches were conducted to discover their inhibitory potential against IL-8 production, iNOS, and COX-2 expression levels by evaluating their binding affinities through binding energies and interactions of ligand-protein residues in the complexes. In the IL-8 model, compounds (**8**, **11**, **16**, and **22**) docked into the IL-8 protein, showing low binding energies ranging from −7.50 to −4.92 kcal/mol (Figure S24, Supplementary Materials). These binding affinities might verify their ability to reduce the level of expression of IL-8 production induced by LPS-stimulated RAW264.7 cells. In the COX-2 model, all the docked compounds showed binding energies ranging from −4.02 to −5.86 kcal/mol (Figure 8). In contrast, these compounds also exhibited docking scores ranging from −8.34 to −6.92 kcal/mol when they were docked to the iNOS protein. Thus, these compounds were prepared for Western blotting assays.

### 3.8. Western Blot Analysis

**Figure 9:**
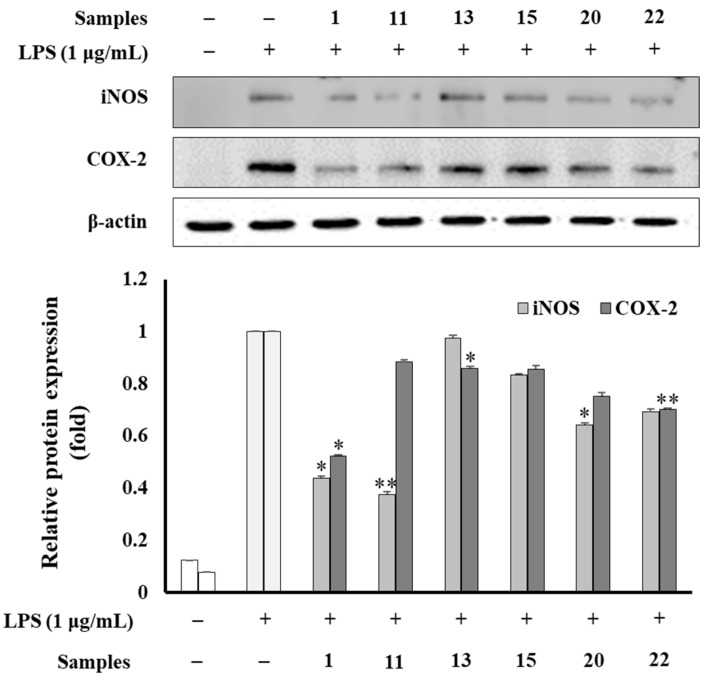
Effects of compounds (**1**, **11**, **13**, **15**, **20**, and **22**) on iNOS and COX-2 expression induced by LPS on RAW264.7 cells. Relative density was calculated as the ratio of the expression level of each protein to β-actin. The data are expressed as the mean ± SD (*n* = 3). * *p* < 0.05, ** *p* < 0.01, compared with the LPS-stimulated group.

Compounds (**1**, **11**, **13**, **15**, **20**, and **22**) suppressed the expression of iNOS and COX-2 enzymatic proteins in LPS-induced RAW264.7 cells by Western blotting assay. Among them, compounds (**1**, **11**, **20**, and **22**) significantly reduced the expression level of both iNOS and COX-2 (Figure 9). Whereas compound **13** showed a weak inhibitory effect. Compound **15** displayed a moderate inhibition of the iNOS expression level and a weak inhibition of the COX-2 expression level.

## 4. Discussion

The combination of an analysis workflow-based molecular network and biological guidance led to the approach of the research object targeting the anti-inflammatory capacity of molecules. in which a high-mass MS/MS-based molecular network has been allowed to identify natural products rapidly. Furthermore, it has been developed and utilized in various fields such as forensic analysis, clinical diagnosis, and the annotation of putative drug metabolites [46,47]. Notably, molecular networks are beneficial for visualization, clustering, comparison, and quantitation, which enable users to recognize the metabolite and inter-sample differences as well as the therapeutic lead discovery of target molecules. We investigated the chemicals from the total extract of different organs of *V. tenuifolia* by analyzing their precursor and parent mass and MS/MS spectral properties using FBMN and NAP through the GNPS web platform. As a result, the flowers of *V. tenuifolia* were selected for research due to their high content of 6′′-acetylapiin compared to other organs of this plant. Subsequently, anti-inflammatory activity guided the establishment of an optimal extraction using variables of organic solvent ratio of MeOH and EtOH in water ranging from 0 to 100%, extraction temperature, and time. The final extraction condition was obtained using 100% EtOH at 25 °C for 90 min by sonification based on the inhibitory effect against NO production in LPS-stimulated RAW264.7 cells. Then, the optimal extract condition was employed to extract the dried flowers of *V. tenuifolia,* which were successfully fractionated into fractions. Above, bioassay-guided extraction and fractionation supported industrial-scale preparation chromatography.

These fractions were evaluated for TPC, TFC, antioxidant, and anti-inflammatory assays. Among them, the Bu fraction showed the highest content of phenolic and flavonoid compounds and the most potent scavenging activity on DPPH and ABTS radicals. As a result, this active fraction was chosen for the isolation and identification of four phenolics, five alkaloids, and 13 flavonoid glycosides from the flowers of *V*. *tenuifolia*. Among isolates, vicia D (**1)** is a new compound, 6-methoxykaempferol 3-*O*-sophoroside (**15)** and diosmetin 7-*O*-(2″-apiosyl)-glucoside (**22**) were first reported NMR data from this study, and ferulic acid (**2**), *trans*-*p*-coumaroyl-D-glucopyranose (**3**), *cis*-*p*-coumaroyl-D-glucopyranose (**4**), L-tryptophan (**5**) and D-tryptophan (**6**), D-phenylalanine (**7**), L-phenylalanine (**8**), adenosine (**9**), afzelin (**10**), rhamnocitrin 3-*O*-glucoside (**11**), kaempferol 7-*O*-glucoside (**12**), kaempferol 3-*O*-(2″-*O*-*β*-D-glucopyranosyl)-*α*-L-rhamnopyranoside (**13**) [30], kaempferol 3-*O*-rutinoside (**14**), quercetin-3-*O*-rhamnoside (**16**), rutin (**17**), rhamnetin 3-*O*-rutinoside (**18**), 6″-acetylapiin (**19**), graveobioside A (**20**), and diosmetin 7-*O*-rutinoside (**21**), were isolated for the first time from this plant. All the isolated compounds exhibited some significant inhibition against inflammatory cytokines and mediators. Briefly, isolates (**1**–**22**) showed an inhibitory effect against NO production without a cytotoxic effect at a tested concentration of 100 μM. Whereas, compounds **5**, **13**, **21**, and **22** displayed a moderate to strong inhibitory effect against IL-1β production at the tested conditions. A structural activity relationship was concluded based on the IL-1β assay. Compounds (**10**–**14**) belonged to kaemferol glycosides. However, compound **13** showed the strongest inhibition against IL-1β production compared to others. This observation suggested that the rhamnosidation at C-3 might be favorable to inhibiting IL-1β production. Compounds (**20**–**22**) shared the same luteolin glycosides. However, compounds (**21** and **22**) have an OCH_3_ attached B-ring, showing a strong anti-inflammatory effect. Thus, this functional group is important for inhibiting IL-1β production. In contrast, compounds (**8**, **11**, **16**, and **22**) significantly inhibited IL-8 production at concentrations of 100 μM. A structural activity relationship was concluded based on the IL-8 assay. Compounds (**10**–**14**) possess the same kaemferol backbone. However, compound **11** showed the strongest inhibition against IL-8 production compared to others. Thus, the glucosidation at C-3 might be favorable to inhibiting IL-8 production. Compounds (**16**–**18**) have the same quercetin backbone. Compound **16** contained 3-rhamnoside, showing a better inhibitory effect than those of **17** and **18**. Therefore, rhamnosidation at C-3 may promote an inhibitory effect against IL-8 production. Compounds (**20**–**22**) shared the same luteolin glycosides. However, compound **22** has 3′-OCH_3_, showing the best anti-inflammatory effect. Thus, this functional group may be a key promoter of inhibitory effects against IL-8 production. Interestingly, this is the first time that compounds (**4**, **13**, **15**, **20**, and **22**) and (**3**, **4**, **11**, **13**, **15**, **18**, **20**, and **22**) have been reported to have inhibitory effects against NO production induced in LPS-stimulated RAW264.7 and LPS-induced IL-8 production in HT-29 cells, respectively. The bioactive constituents (**1**–**22**) were obtained by optimizing the extraction process and chromatographic method with significant inhibition, thus highlighting their own fraction. This finding also clarifies the anti-inflammatory capacity of the total extract of this plant.

Furthermore, the active compounds (**1**, **11**, **13**, **15**, **20**, and **22**) were evaluated for their action mode by Western blotting assay. Among them, compounds (**1**, **11**, **20**, and **22**) showed a significant reduction in the expression of iNOS and COX-2. Therefore, it was hypothesized that these inhibitors would have an impact on NO generation by blocking the catalyst enzymes COX-2 and iNOS during the rate-limiting steps producing PGE2 and NO, respectively. Besides the protective effect on the integrity of the epithelial intestinal wall and suppressing colitis symptoms, prostaglandin E2 (PGE2) exacerbates the inflammatory progression of IBD by switching the cytokines and chemokines profile via different pathways [48]. On the other hand, iNOS and COX-2 enzymes are the key catalysts for producing NO and PGE2. Conversely, PGE2 also upregulates the expression of COX-2. These feedback loops may amplify the activity of both COX-2 and PGE2 during inflammatory progression [49]. Thus, iNOS and COX-2 inhibitors are considered a theory to prevent inflammatory diseases through downregulating PGE2. These active compounds also exhibited low binding energy when they were docked into the iNOS and COX-2 proteins to form the complexes in silico. On the other hand, these compounds also interacted with amino acids in the binding pocket of proteins through hydrogen or hydrophobic interactions, respectively. In addition, in silico studies revealed that compounds (**8**, **11**, **16**, and **22**) exhibited good binding affinity when they were docked into the IL-8 protein with low docked scores. These compounds occurred in the IL-8 protein, surrounded by hydrogen and hydrophobic interactions. In silico results confirmed the biological activity of active compounds against inflammatory cytokines and mediators, suggesting that these active components should be examined for further in vivo studies in order to verify their activity capacity for the purpose of developing products for the treatment of inflammatory diseases.

## 5. Conclusions

This study is the first comprehensive research on the preparation of bioactive fractions and purified compounds from *V. tenuifolia* targeting anti-inflammatory activity. This is the first report on optimization of the extraction process of active components from *V. tenuifolia*, which includes the solvent ratio of the usual organic solvent as well as extraction time and temperature. Bioactive components were found to correspond to the anti-inflammatory potential of the plant. These active compounds were also shown to downregulate the enzymatic proteins (iNOS and COX-2) during the production of the inflammatory mediators, and they also inhibited cytokine (IL-8 and IL-1β) production in inflammatory progression. Our in vitro and docking studies revealed significant potential for the anti-inflammatory capacity of active components by accessing their experimental and in silico models. The above optimal extract and fractionation results also demonstrated that the active fraction had antioxidant capability and a good concentration of phenolic and flavonoid components. Therefore, the existence of the bioactive components indicates that this legume may support advancements in industrial food areas for human health against inflammation. This was the first primary study reporting on the anti-inflammatory properties of fractions and compounds, which is a newly reported benefit of *V. tenuifolia*. The knowledge gained from this study provides new insights into the great potential of this legume in functional product development due to its medicinal value. This is also proof that such legumes are not only rich in various nutrients, but it also broadens the knowledge of their bioactivities.

## Figures and Tables

**Table 1:** ^1^H NMR (400 MHz) and ^13^C NMR (100 MHz) spectroscopic data for compounds **1**, **15**, and **22**, acquired in DMSO-*d*_6_ [*δ*_H_, multiplicity (*J* in Hz)].

Position	1		15		22	
1	-	107.5	-	-	-	-
2	-	157.0	-	158.7	-	164.6
3	6.50 (1H, d, 2.3)	101.9	-	134.9	6.84 (1H, s)	103.6
4	-	157.3	-	179.9	-	179.8
5	6.21 (1H, d, 2.3)	104.0	-	150.4	-	160.8
6	-	150.3	-	129.0	6.44 (1H, brs)	97.8
7	3.45 (1H, d, 17.0) 3.62 (1H, d, 17.0)	29.0	6.24 (1H, s)	158.1	-	163.0
8	-	169.3	-	100.0	6.79 (1H, brs)	94.5
9	-	-	-	158.4	-	156.9
10	-	-	-	104.8	-	105.1
1′	-	117.9	-	122.9	-	122.8
2′	7.11 (1H, d, 1.9)	105.2	8.09 (1H, d, 8.9)	132.3	7.46 (1H, d, 2.4)	112.9
3′	-	148.0	6.90 (1H, d, 8.9)	116.3	-	146.6
4′	-	140.3	-	161.7	-	151.3
5′	-	145.5	6.90 (1H, d, 8.9)	116.3	7.10 (1H, d, 8.6)	111.9
6′	7.16 (1H, d, 1.9)	111.1	8.09 (1H, d, 8.9)	132.3	7.57 (1H, dd, 2.4, 8.6)	118.6
7′	-	163.8	-	-	-	-
OCH_3_	3.78 (3H, s)	55.9	3.87 (3H, s)	61.9	3.87 (3H, s)	55.5
Glucosyl	2-*O*-Glc		3-*O*-Glc		7-*O*-Glc	
1″	4.66 (1H, d, 7.3)	103.4	5.45 (1H, d, 7.6)	100.9	5.18 (1H, d, 7.3)	99.1
2″	3.20 (m)	73.2	3.74 (1H, m)	82.6	3.53 (1H, m)	76.5
3″	3.29 (m)	77.0	3.59 ((1H, d, 8.9)	77.9	3.50 (1H, m)	76.8
4″	3.15 (m)	69.3	3.35 (overlap)	71.3	3.20 (1H, m)	69.5
5″	3.22 (m)	76.3	3.18 (1H, m)	78.3	3.43 (1H, m)	75.4
6″	3.44 (m) 3.67 (m)	60.6	3.47 (1H, dd, 5.7, 11.9) 3.70 (1H, m)	62.6	3.45 (1H, m) 3.73 (1H, m)	60.3
Glycosyl	8-*O*-Rha		2″-*O*-Glc		2″-*O*-Api	
1‴	5.65 (1H, d, 1.8)	94.0	4.74 (1H, d, 7.4)	104.8	5.35 (1H, d, 1.5)	108.5
2‴	3.49 (m)	69.4	3.36 (overlap)	75.6	3.74 (1H, d, 1.5)	75.8
3‴	3.23 (m)	70.1	3.35 (overlap)	77.9	-	78.9
4‴	3.15 (m)	71.4	3.33 (overlap)	71.1	3.66 (1H, d, 9.4) 3.91 (1H, d, 9.4)	73.8
5‴	3.29 (m)	70.7	3.28 (overlap)	78.2	3.31 (overlap)	64.0
6‴	0.96 (1H, d, 6.2)	17.7	3.30 3.35 (overlap) 3.65 (1H, m)	62.4	-	-

Assignments were confirmed by COSY, HSQC, HMBC, and NOESY spectra.

## Data Availability

All of data are contained within the article.

## Supplementary Materials

The following supporting information can be downloaded at: https://www.mdpi.com/article/10.3390/antiox12101876/s1.

## References

[1] Kałużna A., Olczyk P., Komosińska-Vassev K. (2022). The role of innate and adaptive immune cells in the pathogenesis and development of the inflammatory response in ulcerative colitis. J. Clin. Med..

[2] Bamias G., Arseneau K.O., Cominelli F. (2014). Cytokines and mucosal immunity. Curr. Opin. Gastroenterol..

[3] Brynskov J., Nielsen O.H., Ahnfelt-Rønne I., Bendtzen K. (2008). Cytokines (immunoinflammatory hormones) and their natural regulation in inflammatory bowel disease (Crohn’s Disease and Ulcerative Colitis): A review. Dig. Dis..

[4] Sanchez-Munoz F., Dominguez-Lopez A., Yamamoto-Furusho J.-K. (2008). Role of cytokines in inflammatory bowel disease. World J. Gastroenterol..

[5] Ley S.H., Hamdy O., Mohan V., Hu F.B. (2014). Prevention and management of type 2 diabetes: Dietary components and nutritional strategies. Lancet.

[6] Bazzano L.A., Thompson A.M., Tees M.T., Nguyen C.H., Winham D.M. (2011). Non-soy legume consumption lowers cholesterol levels: A meta-analysis of randomized controlled trials. Nutr. Metab. Cardiovasc. Dis..

[7] Megías C., Cortés-Giraldo I., Girón-Calle J., Alaiz M., Vioque J. (2018). Characterization of Vicia (Fabaceae) seed water extracts with potential immunomodulatory and cell antiproliferative activities. J. Food Biochem..

[8] Pastor-Cavada E., Juan R., Pastor J.E., Alaiz M., Vioque J. (2009). Fatty acid distribution in the seed flour of wild *Vicia* species from southern Spain. J. Am. Oil Chem. Soc..

[9] Pastor-Cavada E., Juan R., Pastor J.E., Alaiz M., GirÓN-Calle J., Vioque J. (2011). Antioxidative activity in the seeds of 28 *vicia* species from southern Spain. J. Food Biochem..

[10] Yang M.H., Ha I.J., Ahn J., Kim C.-K., Lee M., Ahn K.S. (2023). Potential function of loliolide as a novel blocker of epithelial-mesenchymal transition in colorectal and breast cancer cells. Cell. Signal..

[11] Yang J.Y., Sanchez L.M., Rath C.M., Liu X., Boudreau P.D., Bruns N., Glukhov E., Wodtke A., de Felicio R., Fenner A. (2013). Molecular networking as a dereplication strategy. J. Nat. Prod..

[12] Crüsemann M., O’Neill E.C., Larson C.B., Melnik A.V., Floros D.J., da Silva R.R., Jensen P.R., Dorrestein P.C., Moore B.S. (2017). Prioritizing natural product diversity in a collection of 146 bacterial strains based on growth and extraction protocols. J. Nat. Prod..

[13] Woo S., Kang K.B., Kim J., Sung S.H. (2019). Molecular networking reveals the chemical diversity of Selaginellin derivatives, natural phosphodiesterase-4 inhibitors from *Selaginella tamariscina*. J. Nat. Prod..

[14] Olivon F., Apel C., Retailleau P., Allard P.M., Wolfender J.L., Touboul D., Roussi F., Litaudon M., Desrat S. (2018). Searching for original natural products by molecular networking: Detection, isolation and total synthesis of chloroaustralasines. Org. Chem. Front..

[15] Zhang F.-X., Li M., Yao Z.-H., Li C., Qiao L.-R., Shen X.-Y., Yu K., Dai Y., Yao X.-S. (2018). A target and nontarget strategy for identification or characterization of the chemical ingredients in Chinese herb preparation Shuang-Huang-Lian oral liquid by ultra-performance liquid chromatography–quadrupole time-of-flight mass spectrometry. Biomed. Chromatogr..

[16] Le D., Han S., Ahn J., Yu J., Kim C.-K., Lee M. (2022). Analysis of Antioxidant Phytochemicals and Anti-Inflammatory Effect from Vitex rotundifolia L.f. Antioxidants.

[17] Le D.D., Min K.H., Lee M. (2023). Antioxidant and anti-Inflammatory capacities of fractions and constituents from *Vicia tetrasperma*. Antioxidants.

[18] Kim C.-K., Yu J., Le D., Han S., Yu S., Lee M. (2023). Anti-inflammatory activity of caffeic acid derivatives from Ilex rotunda. Int. Immunopharmacol..

[19] Le D., Han S., Min K.H., Lee M. (2023). Anti-Inflammatory Activity of Compounds Derived from Vitex rotundifolia. Metabolites.

[20] Li X., Zhang J.-Y., Gao W.-Y., Wang Y., Wang H.-Y., Cao J.-G., Huang L.-Q. (2012). Chemical composition and anti-inflammatory and antioxidant activities of eight pear cultivars. J. Agric. Food Chem..

[21] Shraim A.M., Ahmed T.A., Rahman M.M., Hijji Y.M. (2021). Determination of total flavonoid content by aluminum chloride assay: A critical evaluation. LWT.

[22] Le D.D., Han S., Yu J., Ahn J., Kim C.-K., Lee M. (2023). Iridoid derivatives from Vitex rotundifolia L. f. with their anti-inflammatory activity. Phytochemistry.

[23] Azizah M., Pripdeevech P., Thongkongkaew T., Mahidol C., Ruchirawat S., Kittakoop P. (2020). UHPLC-ESI-QTOF-MS/MS-Based Molecular Networking Guided Isolation and Dereplication of Antibacterial and Antifungal Constituents of Ventilago denticulata. Antibiotics.

[24] Slimestad R., Fossen T., Brede C. (2020). Flavonoids and other phenolics in herbs commonly used in Norwegian commercial kitchens. Food Chem..

[25] Chang S.W., Du Y.E., Qi Y., Lee J.S., Goo N., Koo B.K., Bae H.J., Ryu J.H., Jang D.S. (2019). New Depsides and Neuroactive Phenolic Glucosides from the Flower Buds of Rugosa Rose (*Rosa rugosa*). J. Agric. Food Chem..

[26] Wang H., Du Y.-J., Song H.-C. (2010). α-Glucosidase and α-amylase inhibitory activities of guava leaves. Food Chem..

[27] Iwashina T., Yamaguchi M.-a., Nakayama M., Onozaki T., Yoshida H., Kawanobu S., Ono H., Okamura M. (2010). Kaempferol glycosides in the flowers of Carnation and their contribution to the Creamy white flower color. Nat. Prod. Commun..

[28] Lee Y.-S., Kim S.-H., Yuk H.J., Lee G.-J., Kim D.-S. (2018). Tetragonia tetragonoides (Pall.) Kuntze (New Zealand Spinach) Prevents Obesity and Hyperuricemia in High-Fat Diet-Induced Obese Mice. Nutrients.

[29] Park Y., Moon B.-H., Yang H., Lee Y., Lee E., Lim Y. (2007). Complete assignments of NMR data of 13 hydroxymethoxyflavones. Magn. Reson. Chem..

[30] Liu G., Zhuang L., Song D., Lu C., Xu X. (2017). Isolation, purification, and identification of the main phenolic compounds from leaves of celery (*Apium graveolens* L. var. dulce Mill./Pers.). J. Sep. Sci..

[31] Pinheiro P.G., Santiago G.M.P., da Silva F.E.F., de Araújo A.C.J., de Oliveira C.R.T., Freitas P.R., Rocha J.E., Neto J.B.d.A., da Silva M.M.C., Tintino S.R. (2022). Ferulic acid derivatives inhibiting Staphylococcus aureus tetK and MsrA efflux pumps. Biotechnol. Rep..

[32] Johnsson P., Peerlkamp N., Kamal-Eldin A., Andersson R.E., Andersson R., Lundgren L.N., Åman P. (2002). Polymeric fractions containing phenol glucosides in flaxseed. Food Chem..

[33] Rasmussen S., Wolff C., Rudolph H. (1996). 4′-*O*-*β*-d-glucosyl-cis-p-coumaric acid—A natural constituent of *Sphagnum fallax* cultivated in bioreactors. Phytochemistry.

[34] Lee M., Phillips R.S. (1992). Fluorine substituent effects for tryptophan in 13C nuclear magnetic resonance. Magn. Reson. Chem..

[35] Choi J.-d., Kim J.-H., Lee J.H., Young H.S., Lee T.-S. (1992). Isolation of adenosine and free amino acid composition from the leaves of *Allium tuberosum*. J. Korean Soc. Food Sci. Nutr..

[36] Kim S.C., Moon M.i., Lee H.a., Kim J., Chang M., Cha J. (2019). Skin care benefits of bioactive compounds isolated from *Zanthoxylum piperitum* DC. (Rutaceae). Trop. J. Pharm. Res..

[37] Nishina A., Itagaki M., Suzuki Y., Koketsu M., Ninomiya M., Sato D., Suzuki T., Hayakawa S., Kuroda M., Kimura H. (2017). Effects of Flavonoids and Triterpene Analogues from Leaves of Eleutherococcus sieboldianus (Makino) Koidz. ‘Himeukogi’ in 3T3-L1 Preadipocytes. Molecules.

[38] Pereira C., Barreto Júnior C.B., Kuster R.M., Simas N.K., Sakuragui C.M., Porzel A., Wessjohann L. (2012). Flavonoids and a neolignan glucoside from Guarea macrophylla (Meliaceae). Química Nova.

[39] Hasler A., Gross G.-A., Meier B., Sticher O. (1992). Complex flavonol glycosides from the leaves of Ginkgo biloba. Phytochemistry.

[40] Hou W.-C., Lin R.-D., Lee T.-H., Huang Y.-H., Hsu F.-L., Lee M.-H. (2005). The phenolic constituents and free radical scavenging activities of Gynura formosana Kiamnra. J. Sci. Food Agric..

[41] Hur J.-M., Park J.-C., Hwang Y.-H. (2001). Aromatic acid and flavonoids from the leaves of *Zanthoxylum piperitum*. Nat. Prod. Sci..

[42] Cedeño H., Espinosa S., Andrade J.M., Cartuche L., Malagón O. (2019). Novel Flavonoid Glycosides of Quercetin from Leaves and Flowers of Gaiadendron punctatum G.Don. (Violeta de Campo), used by the Saraguro Community in Southern Ecuador, Inhibit α-Glucosidase Enzyme. Molecules.

[43] Merina A.J., Kesavan D., Sulochana D. (2011). Isolation and antihyperglycemic activity of flavonoid from flower petals of *Opuntia stricta*. Pharm. Chem. J..

[44] Materska M., Piacente S., Stochmal A., Pizza C., Oleszek W., Perucka I. (2003). Isolation and structure elucidation of flavonoid and phenolic acid glycosides from pericarp of hot pepper fruit *Capsicum annuum* L.. Phytochemistry.

[45] Siciliano T., De Tommasi N., Morelli I., Braca A. (2004). Study of flavonoids of *Sechium edule* (Jacq) Swartz (Cucurbitaceae) different edible organs by liquid chromatography photodiode array mass spectrometry. J. Agric. Food Chem..

[46] Quinn R.A., Nothias L.-F., Vining O., Meehan M., Esquenazi E., Dorrestein P.C. (2017). Molecular networking as a drug discovery, drug Metabolism, and precision medicine strategy. Trends Pharmacol. Sci..

[47] Allard S., Allard P.-M., Morel I., Gicquel T. (2019). Application of a molecular networking approach for clinical and forensic toxicology exemplified in three cases involving 3-MeO-PCP, doxylamine, and chlormequat. Drug Test. Anal..

[48] Sheibanie A.F., Yen J.-H., Khayrullina T., Emig F., Zhang M., Tuma R., Ganea D. (2007). The proinflammatory effect of prostaglandin E2 in experimental inflammatory bowel disease is mediated through the IL-23→IL-17 axis. J. Immunol..

[49] Greenhough A., Smartt H.J.M., Moore A.E., Roberts H.R., Williams A.C., Paraskeva C., Kaidi A. (2009). The COX-2/PGE 2 pathway: Key roles in the hallmarks of cancer and adaptation to the tumour microenvironment. Carcinogenesis.

