# Supplementary material for: Molecular Networking and Bioassay-Guided Preparation and Separation of Active Extract and Constituents from Vicia tenuifolia Roth
Source: Antioxidants (Basel). 2023 Oct 18;12(10):1876. doi: 10.3390/antiox12101876 (PMC10604256; doi:10.3390/antiox12101876)
Supplement: Supplementary file 1 [file antioxidants-12-01876-s001.zip › antioxidants-2626039-supplementary.pdf]

Supporting materials

Molecular networking and bioassay-guided preparation and separation of active extract and constituents from *Vicia tenuifolia* Roth

Duc Dat Le, Soojung Yu, Thinhulinh Dang, and Mina Lee*

College of Pharmacy and Research Institute of Life and Pharmaceutical Sciences, Sunchon National University, 255 Jungangno, Suncheon 57922, Jeonnam, Korea

*Corresponding author: Mina Lee

🖂 E-mail: minalee@scnu.ac.kr; Tel.: +82-61-750-3764; Fax: +82-61-750-3708.

Table of Contents

1. Spectroscopic data of marker compounds (1, 15, and 22): 1

Figure S1. HR-ESI-MS spectra of compound **1** 1

Figure S2. ^{1}H-NMR (400 MHz, DMSO-d_6) spectrum of compound **1** 1

Figure S3. ^{13}C-NMR (100 MHz, DMSO-d_6) spectrum of compound **1** 2

Figure S4. DEPT-NMR (DMSO-d_6) spectrum of compound **1** 2

Figure S5. ^{1}H-^{1}H COSY spectrum of compound **1** 3

Figure S6. ^{1}H-^{13}C HMQC spectrum of compound **1** 3

Figure S7. ^{1}H-^{13}C HMBC spectrum of compound **1** 4

Figure S8. ^{1}H-^{1}H NOESY spectrum of compound **1** 4

Figure S9. HR-ESI-MS spectra of compound **15**. 5

Figure S10. ^{1}H-NMR (400 MHz, DMSO-d_6) spectrum of compound **15** 6

Figure S11. ^{13}C-NMR (100 MHz, DMSO-d_6) spectrum of compound **15** 6

Figure S12. ^{1}H-^{1}H COSY spectrum of compound **15** 7

Figure S13. ^{1}H-^{13}C HMQC spectrum of compound **15** 7

Figure S14. ^{1}H-^{13}C HMBC spectrum of compound **15** 8

Figure S15. ^{1}H-^{1}H NOESY spectrum of compound **15** 8

Figure S16. High mass spectrometry of compound **22** 9

Figure S17. ^{1}H-NMR (400 MHz, DMSO-d_6) spectrum of compound **22** 9

Figure S18. DEPT-NMR spectrum of compound **22** 10

Figure S19. ^{1}H-^{1}H COSY spectrum of compound **22** 10

Figure S20. ^{1}H-^{13}C HMQC spectrum of compound **22** 11

Figure S21. ^{1}H-^{13}C HMBC spectrum of compound **22** 11

Figure S22. ^{1}H-^{1}H NOESY spectrum of compound **22** 12

Figure S23. Cell viability of RAW264.7 (**A**) and HT-29 (**B**) by treatment with compounds (**1–22**) ...13

Figure S24. Binding poses and interactions between binding sites of IL-8 receptor with respect to ligands [compounds **8** (**A**), **11** (**B**), **16** (**C**), and **22** (**D**)] 13

1. Spectroscopic and spectrometry data of new compounds (1, 15, and 22):

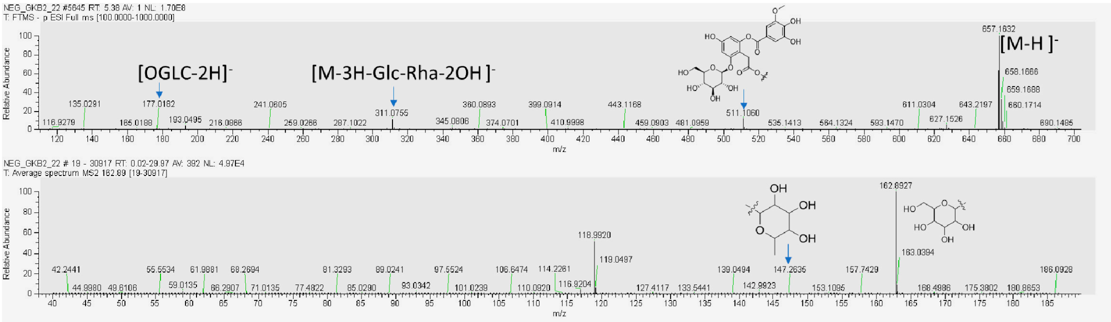

Figure S1. HR-ESI-MS spectra of compound **1**.

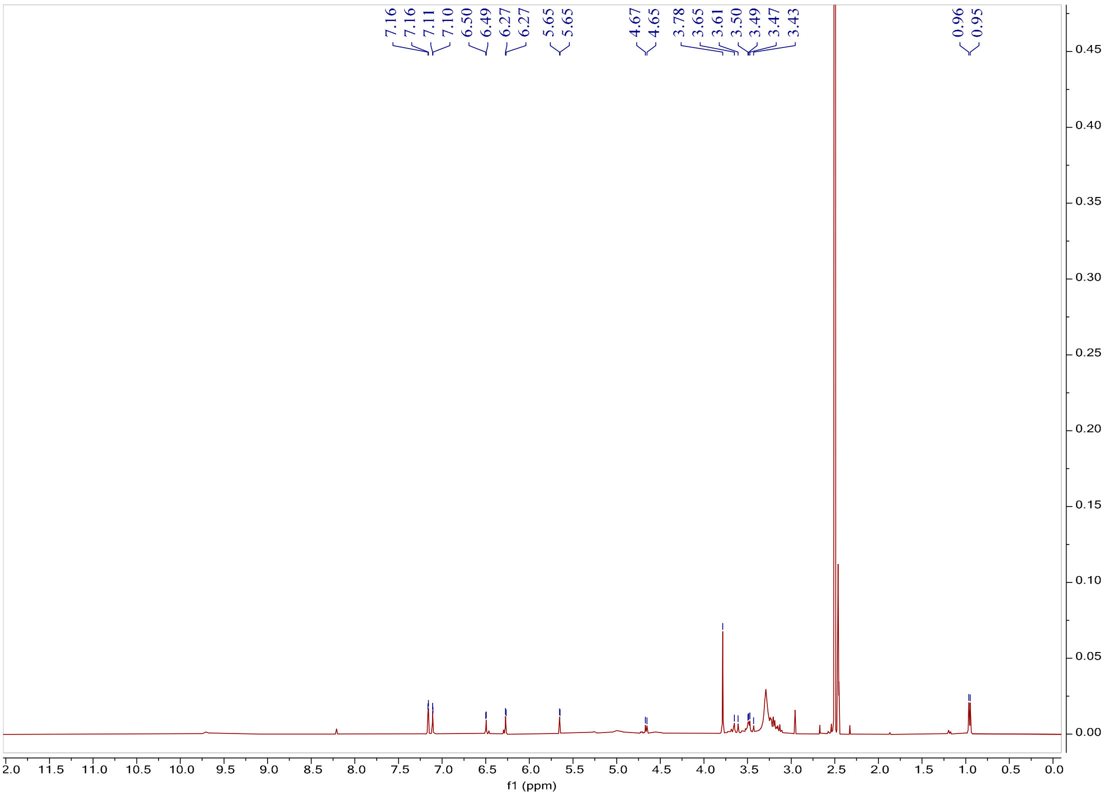

Figure S2. ^{1}H-NMR (400 MHz, DMSO-d_6) spectrum of compound **1**.

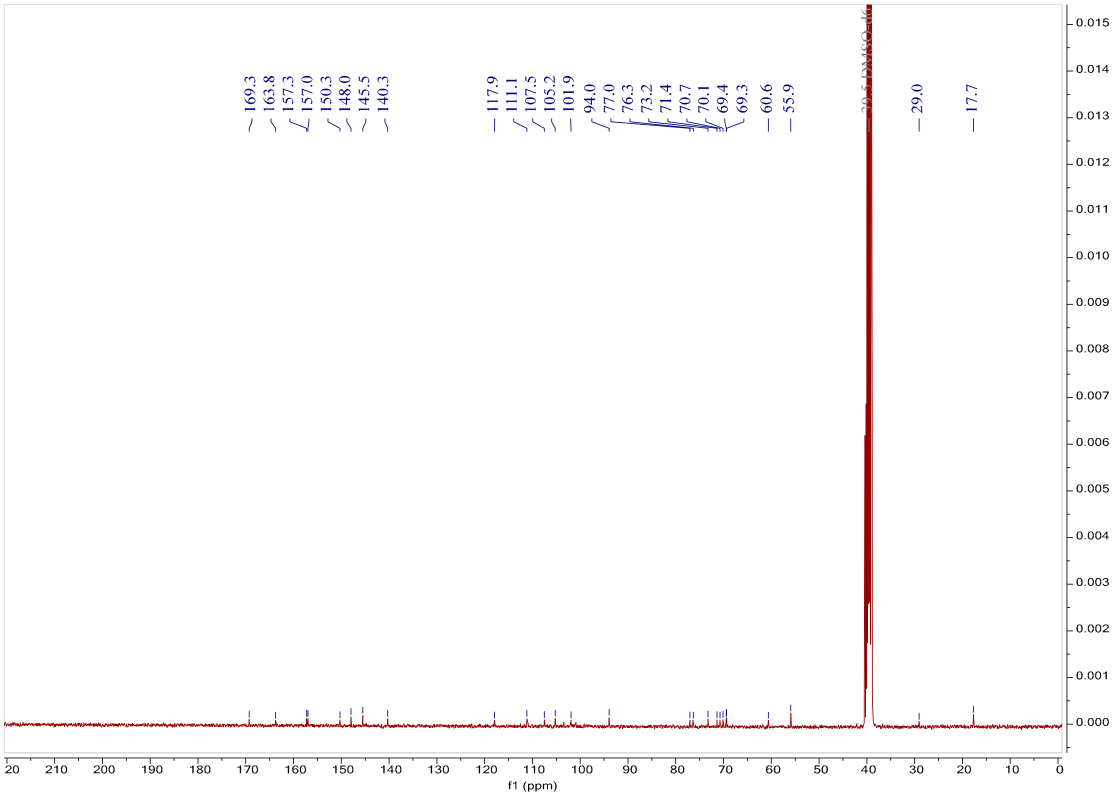

Figure S3. ^{13}C-NMR (100 MHz, DMSO-d_6) spectrum of compound **1**.

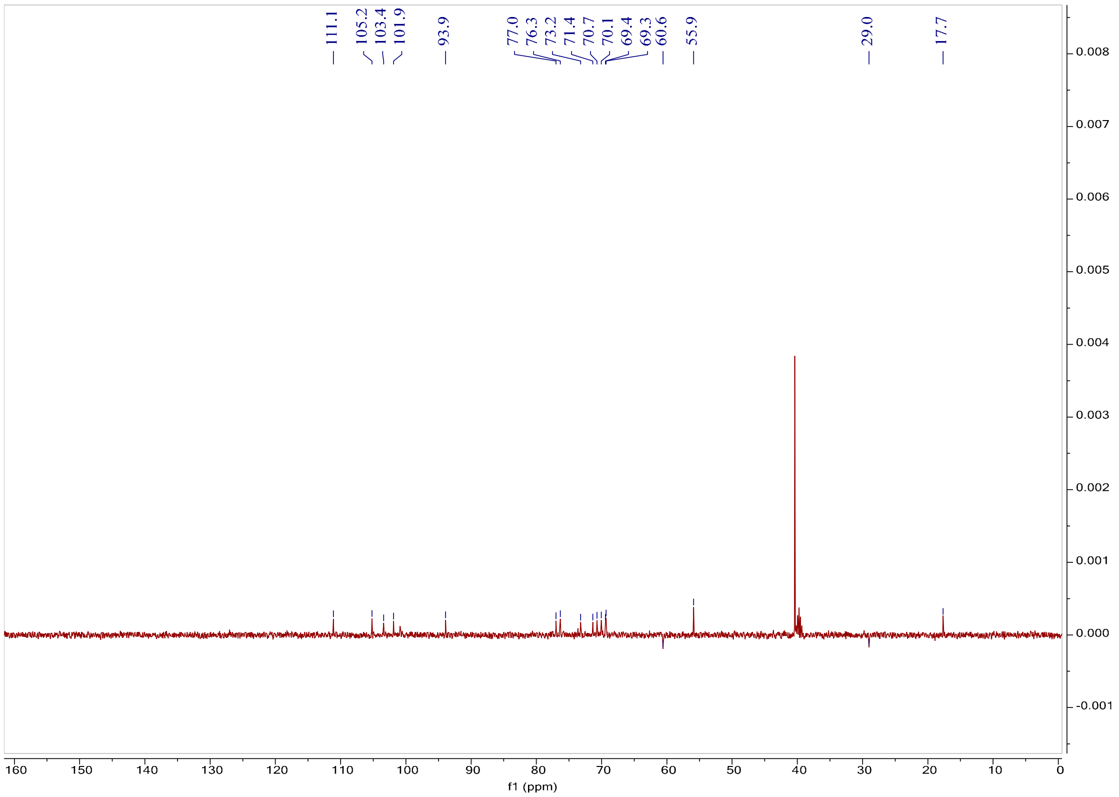

Figure S4. DEPT spectrum of compound 1.

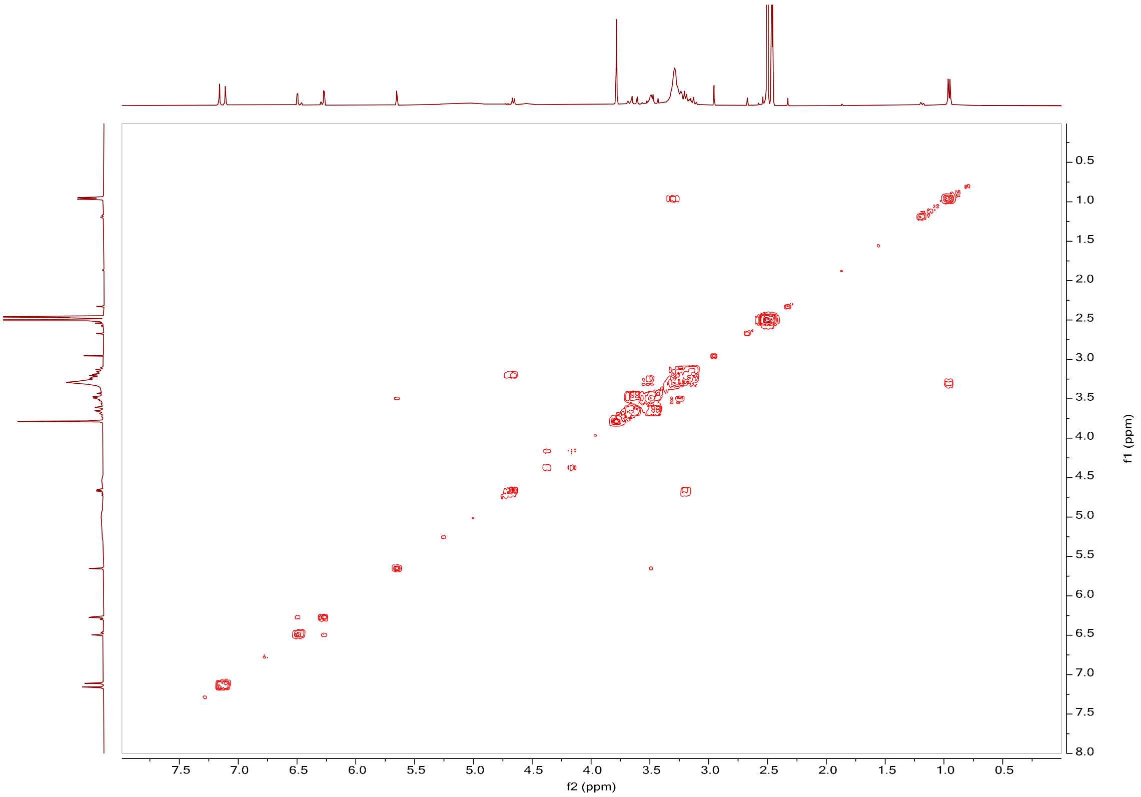

Figure S5. ^{1}H-^{1}H COSY spectrum of compound **1**.

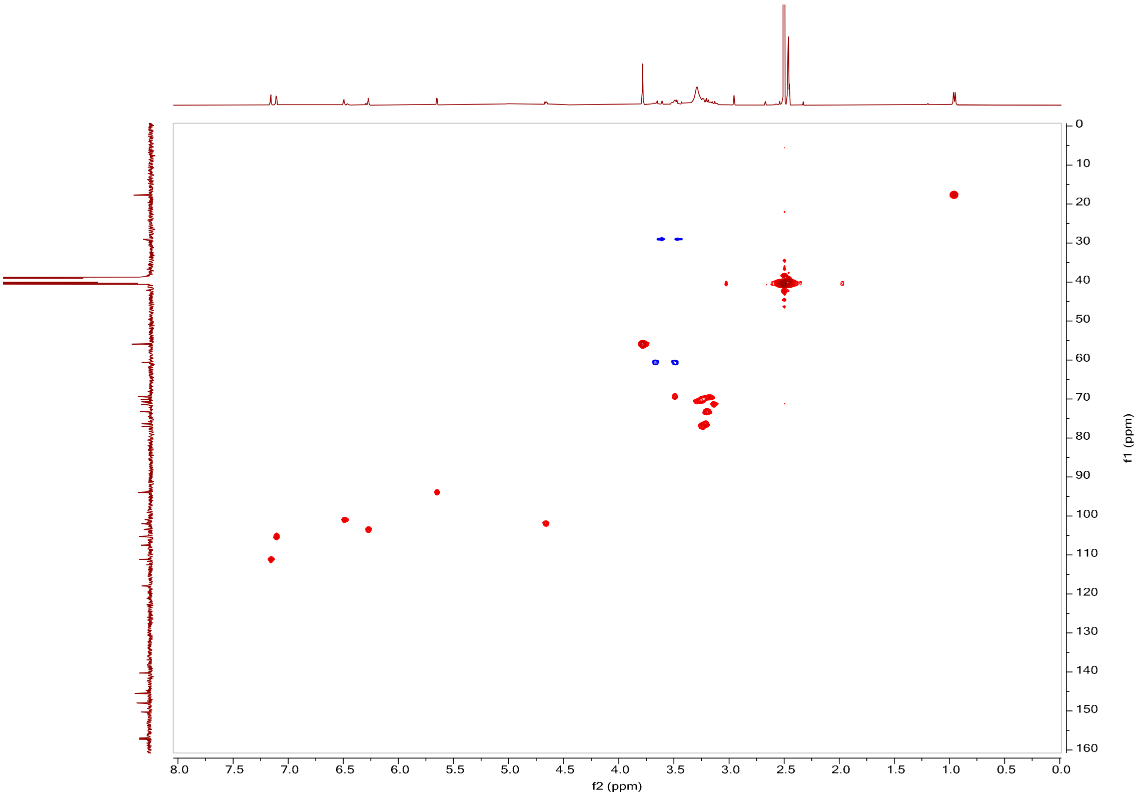

Figure S6. ^{1}H-^{13}C HSQC spectrum of compound **1**.

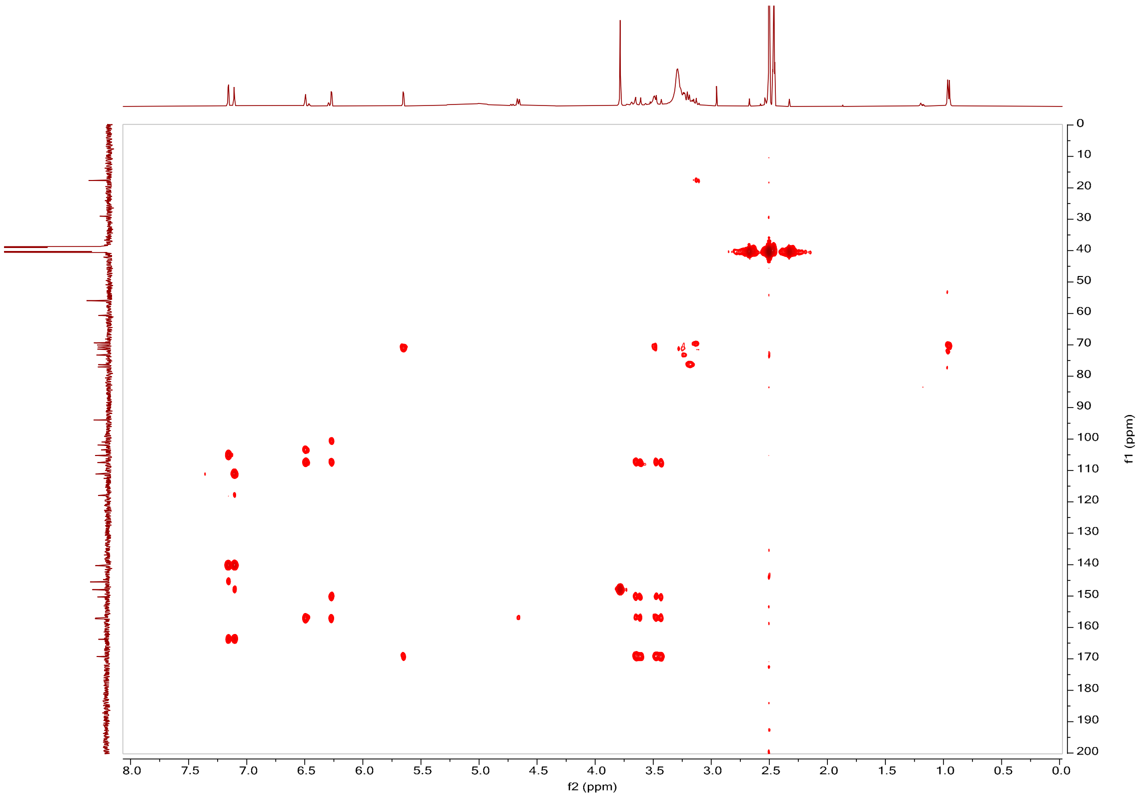

Figure S7. ^{1}H-^{13}C HMBC spectrum of compound **1**.

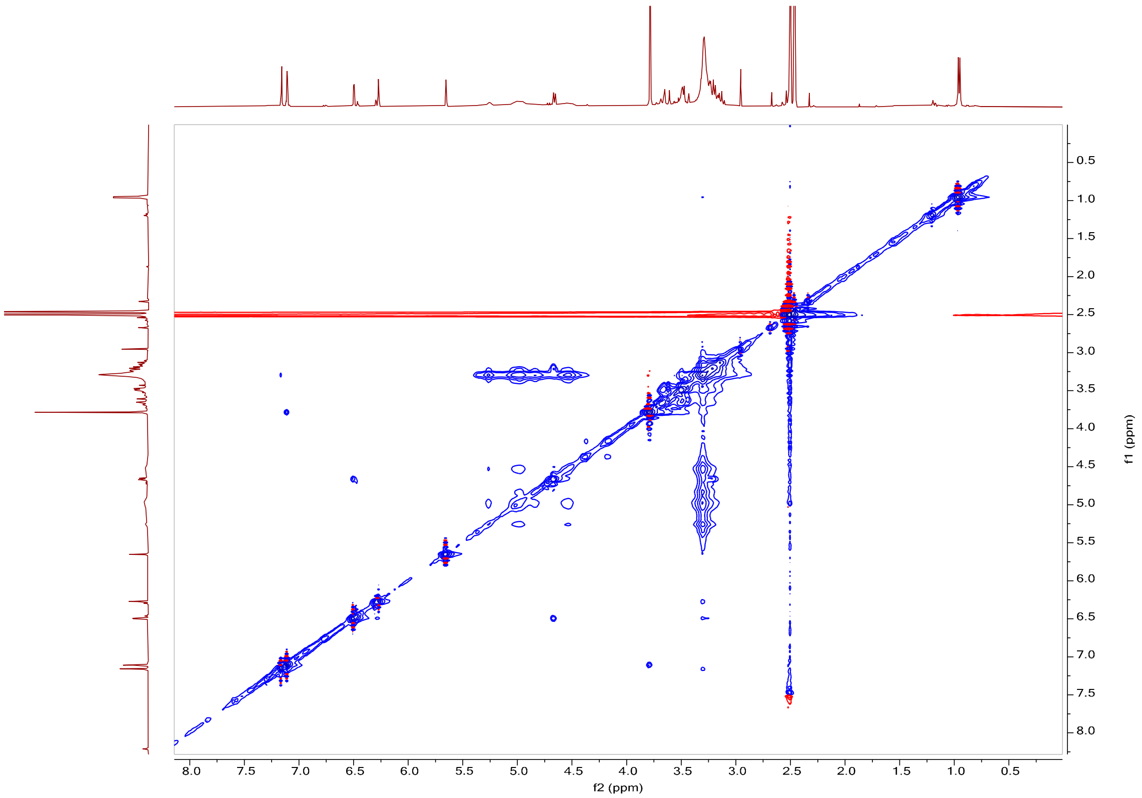

Figure S8. ^{1}H-^{1}H NOESY spectrum of compound **1**.

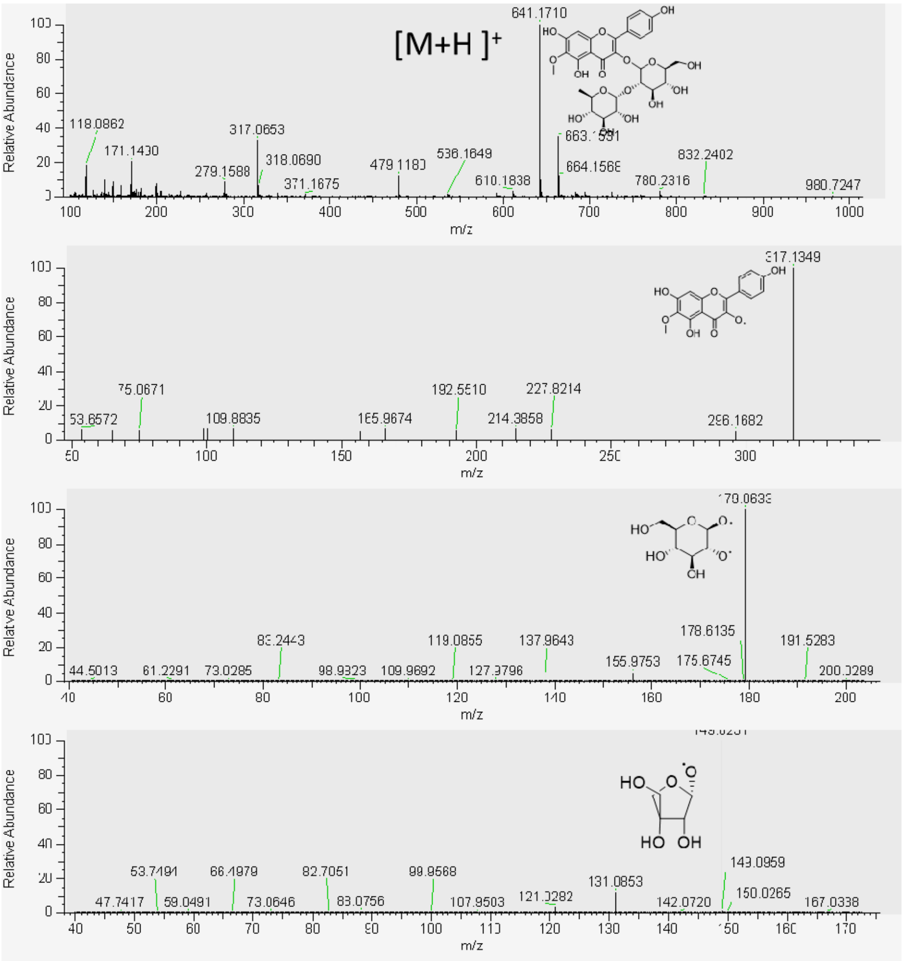

Figure S9. HR-ESI-MS spectra of compound **15**.

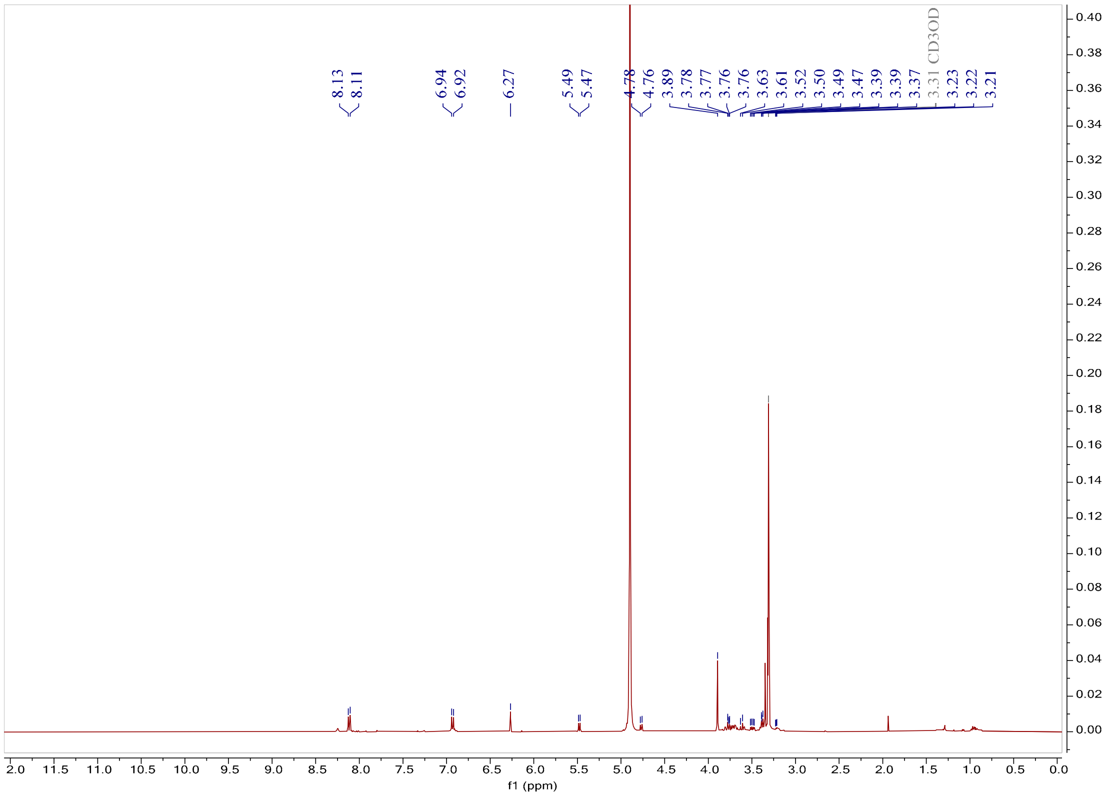

Figure S10. ^{1}H-NMR (400 MHz, DMSO-d_6) spectrum of compound **15**.

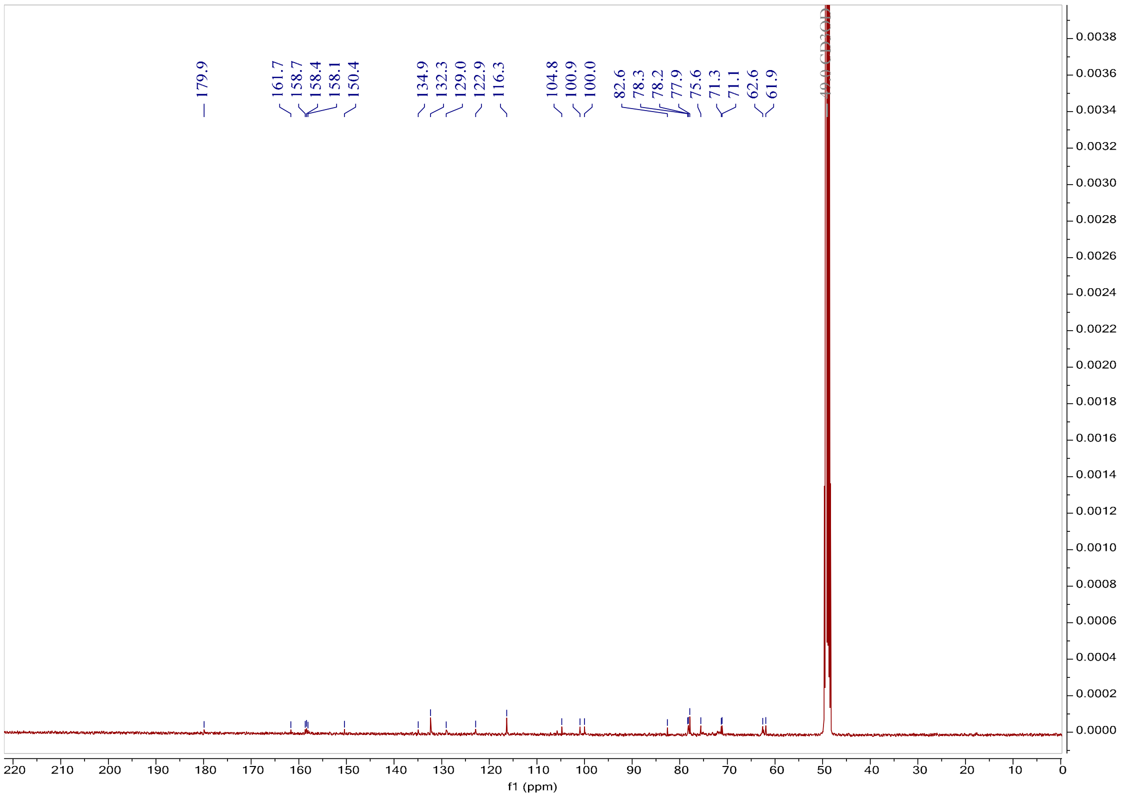

Figure S11. ^{13}C-NMR (100 MHz, DMSO-d_6) spectrum of compound **15**.

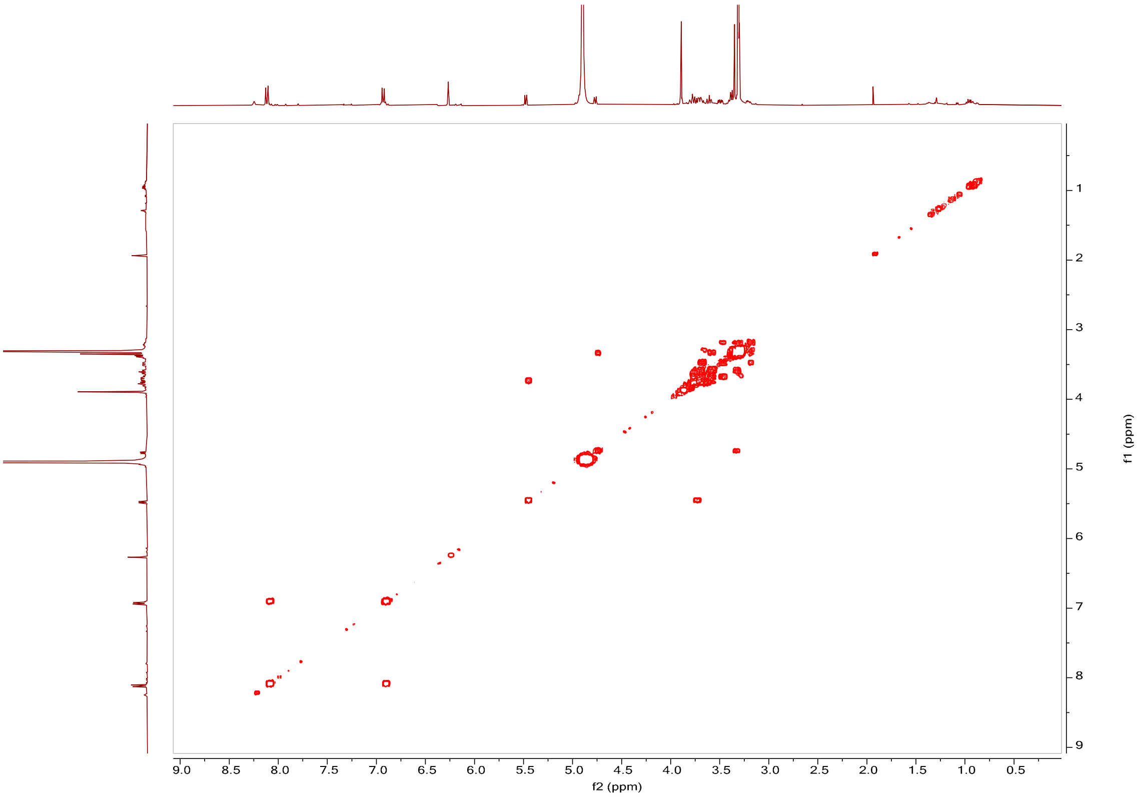

Figure S12. ^{1}H-^{1}H COSY spectrum of compound **15**.

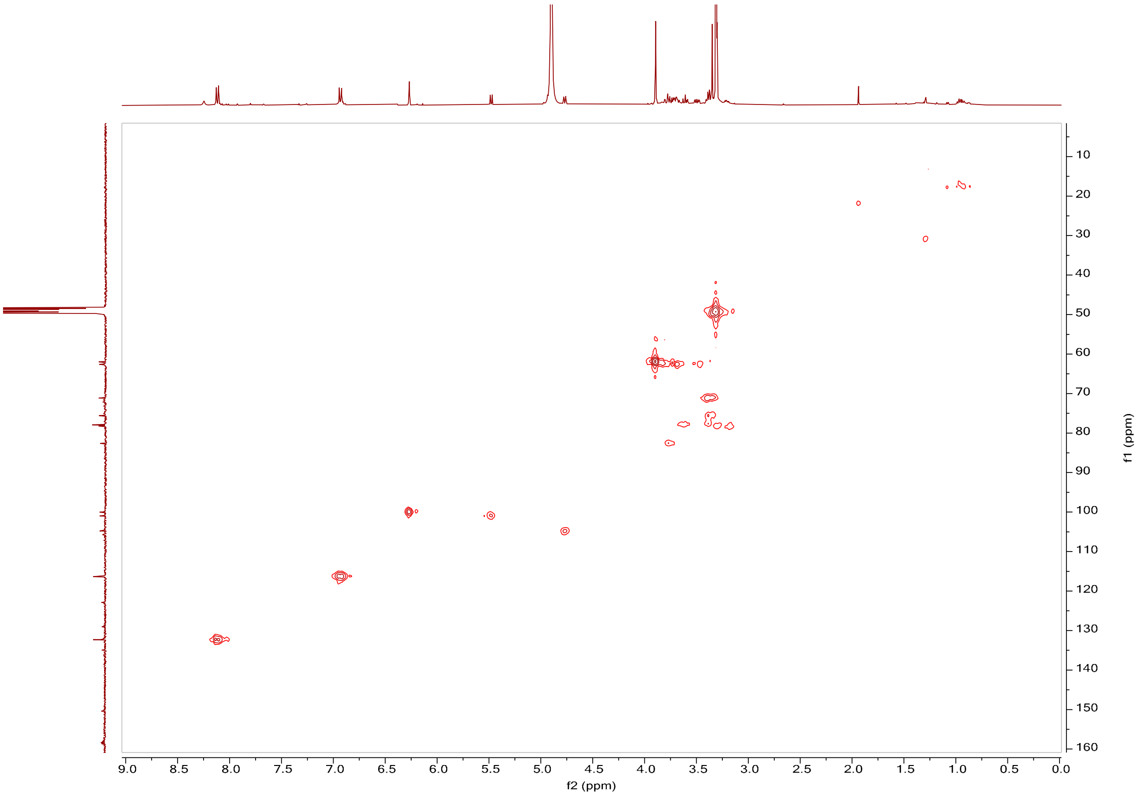

Figure S13. ^{1}H-^{13}C HMQC spectrum of compound **15**.

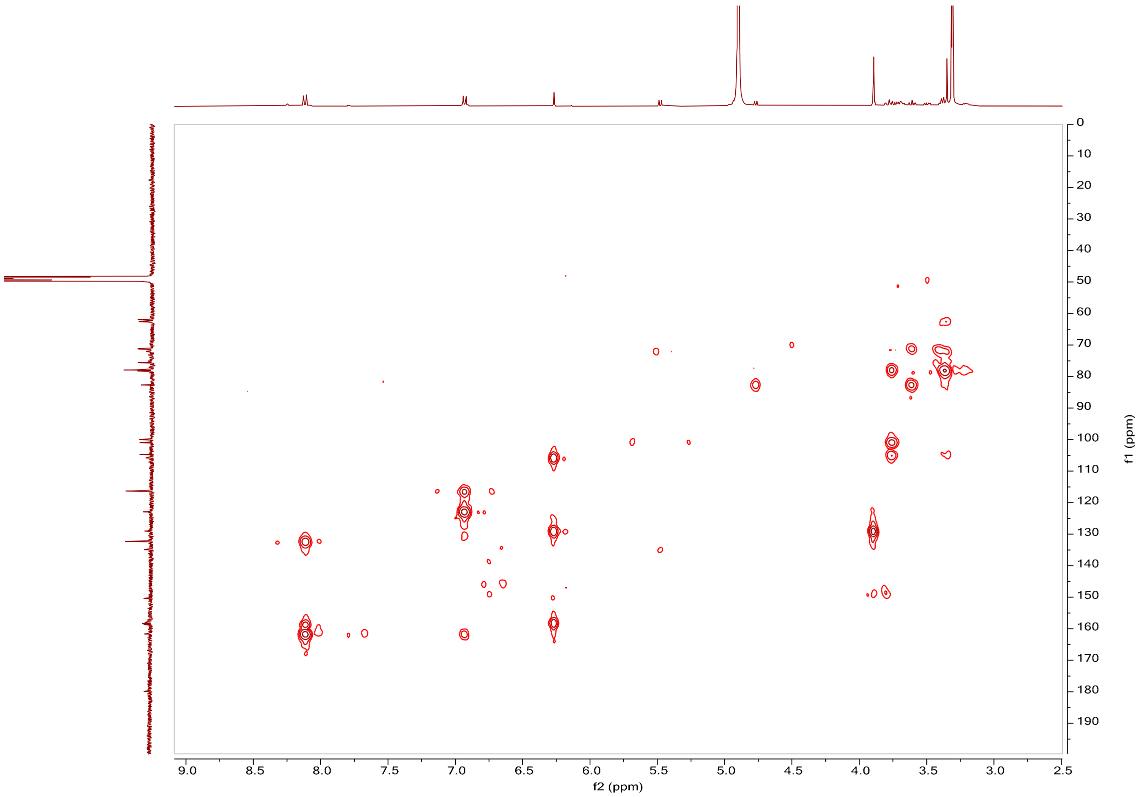

Figure S14. ^{1}H-^{13}C HMBC spectrum of compound **15**.

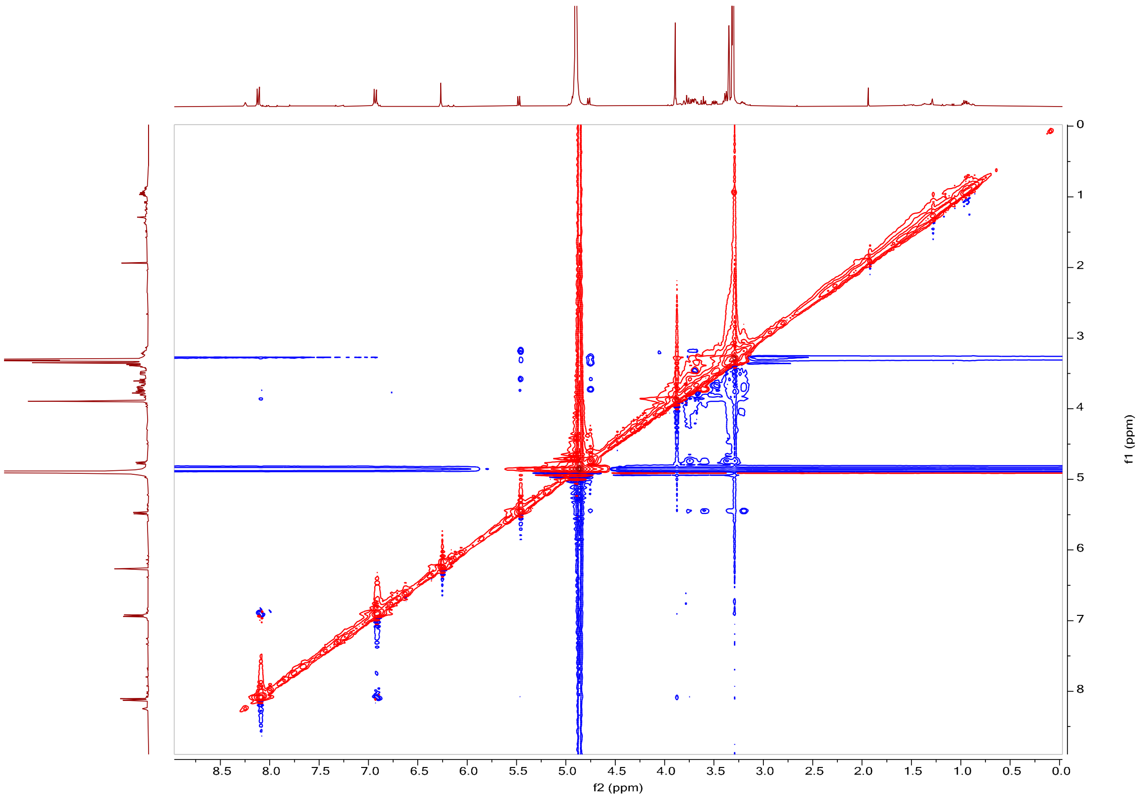

Figure S15. ^{1}H-^{1}H NOESY spectrum of compound **15**.

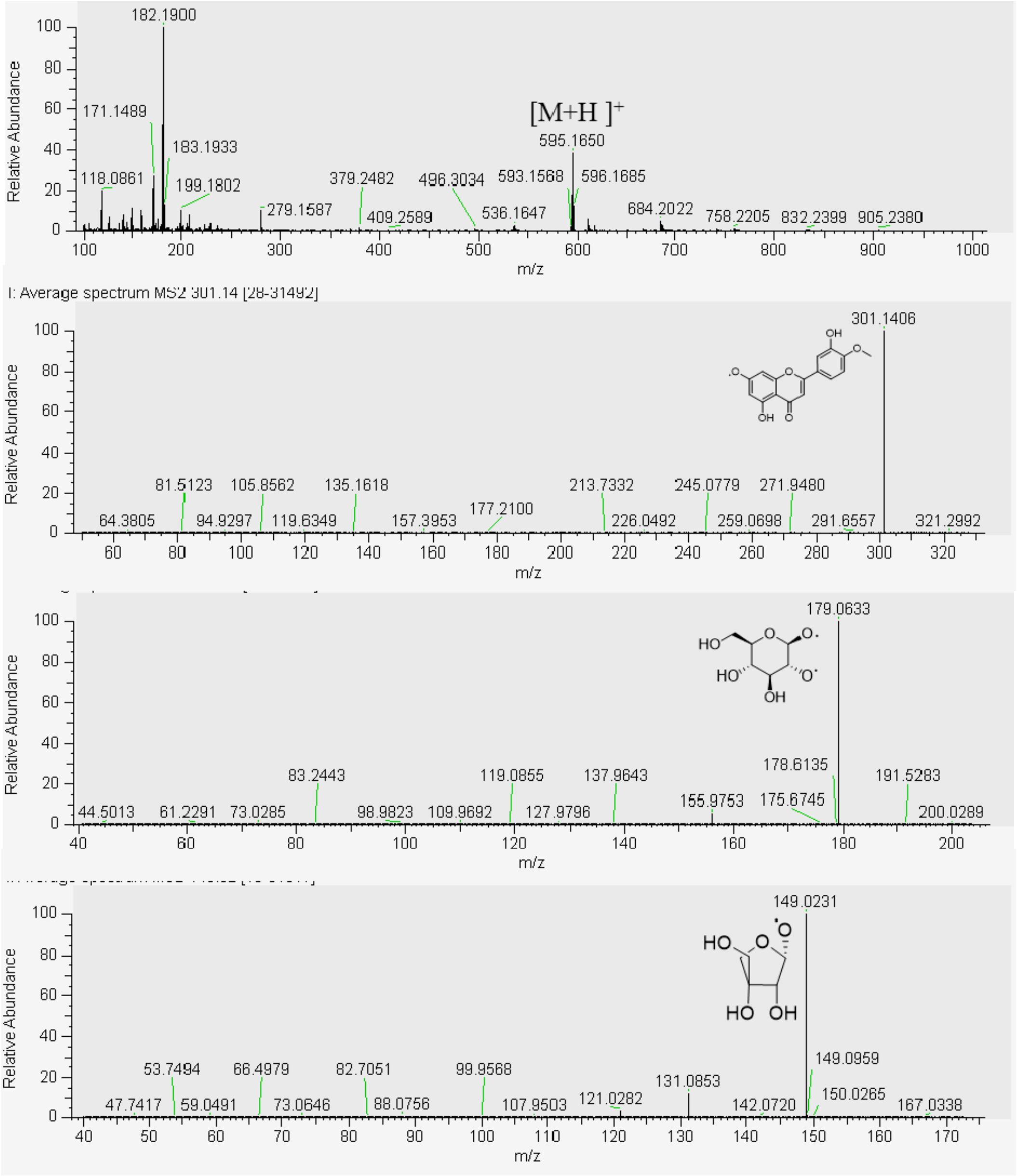

Figure S16. HR-ESI-MS spectra of compound **22**.

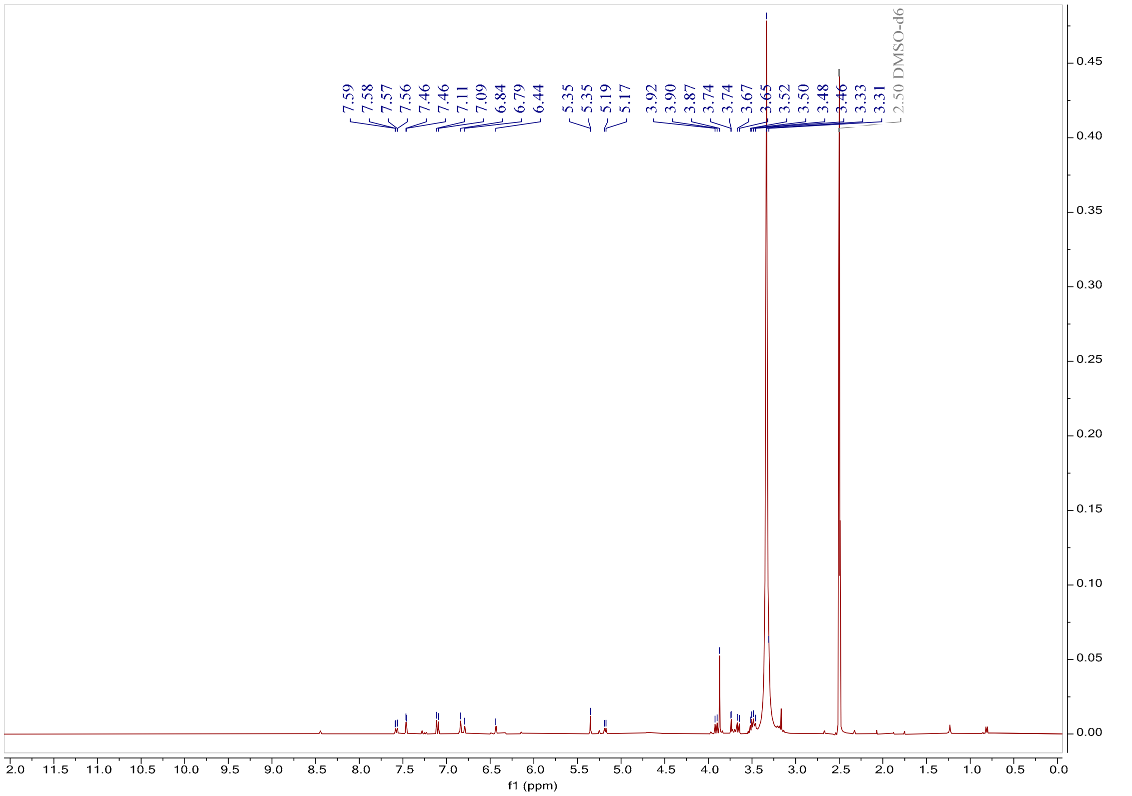

Figure S17. ^{1}H-NMR (400 MHz, DMSO-d_6) spectrum of compound **22**.

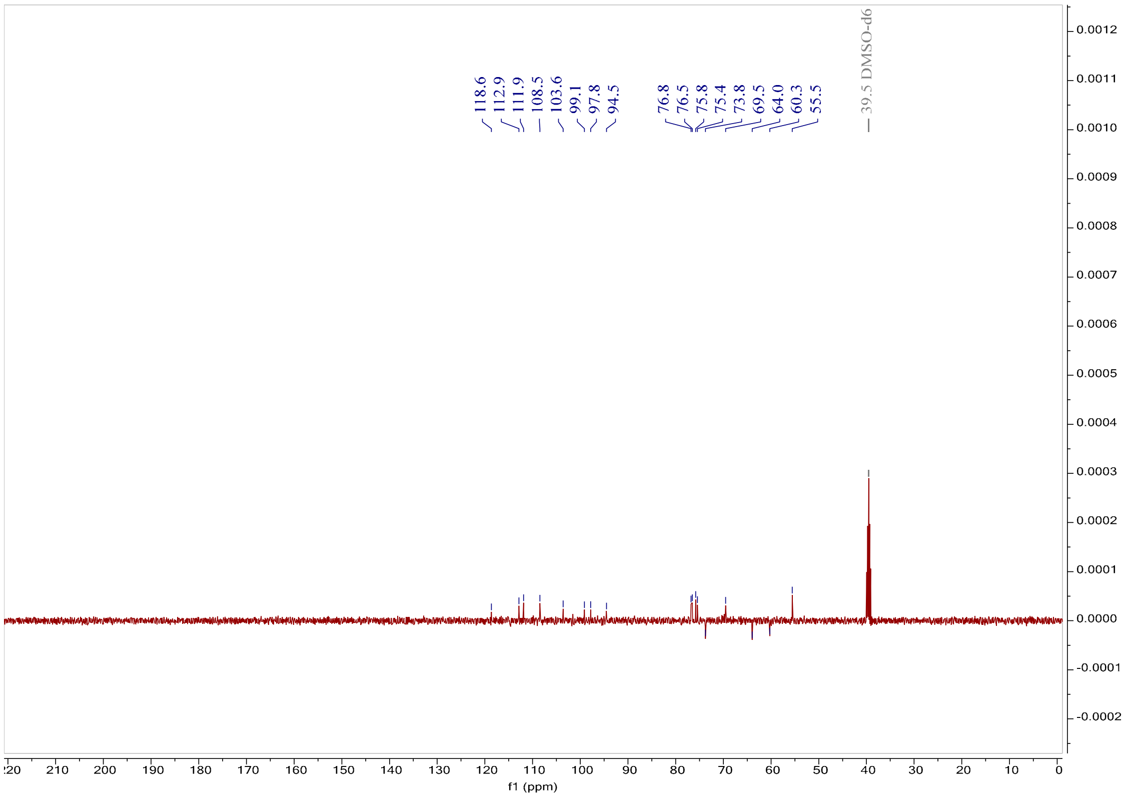

Figure S18. DEPT spectrum of compound **22**.

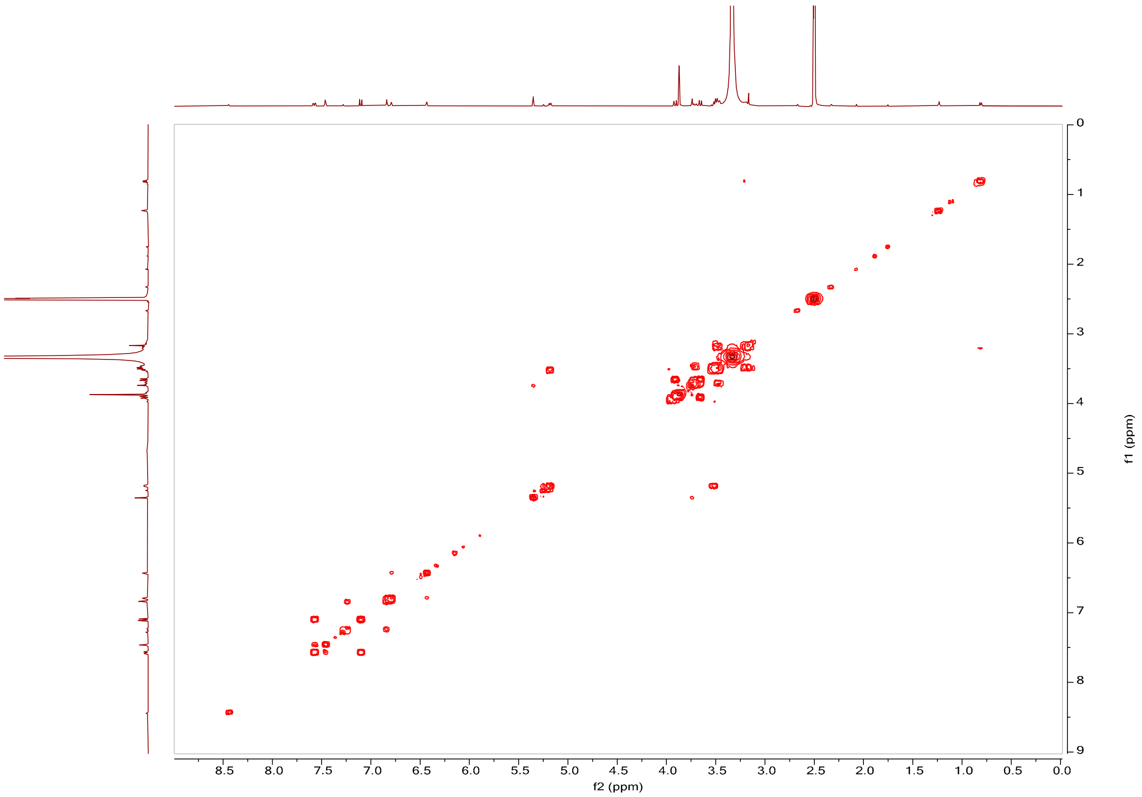

Figure S19. ^{1}H-^{1}H COSY spectrum of compound **22**.

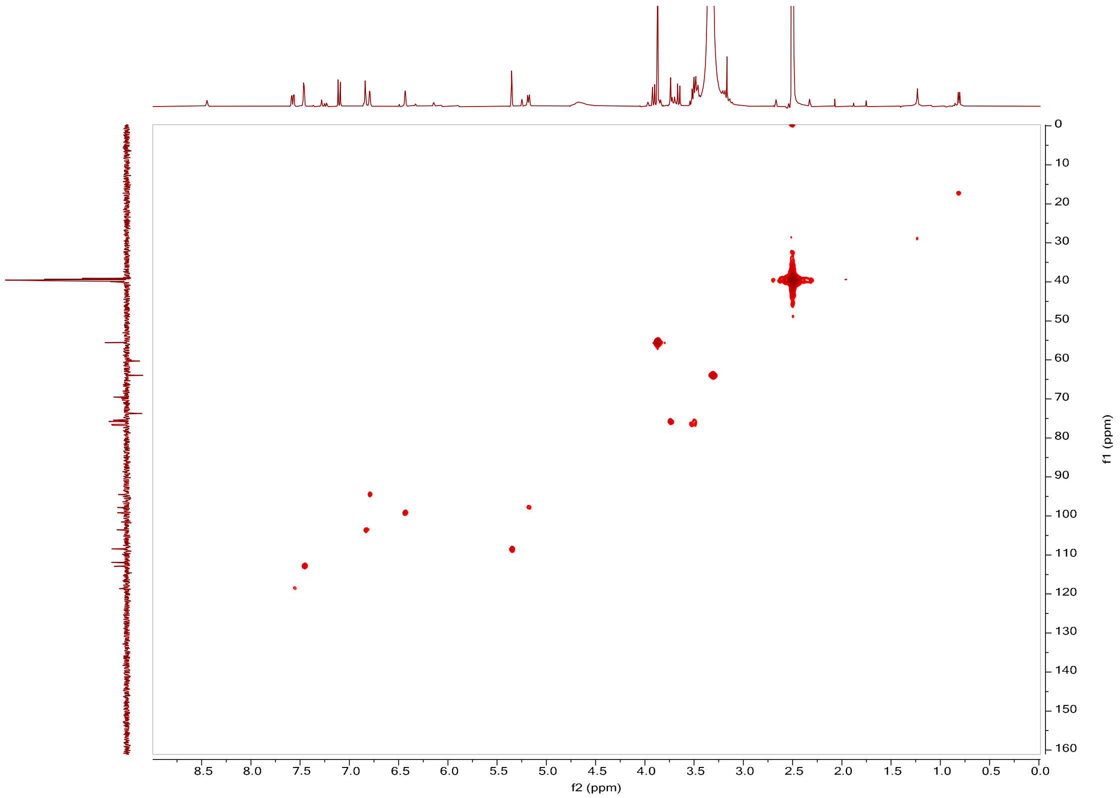

Figure S20. ^{1}H-^{13}C HMQC spectrum of compound **22**.

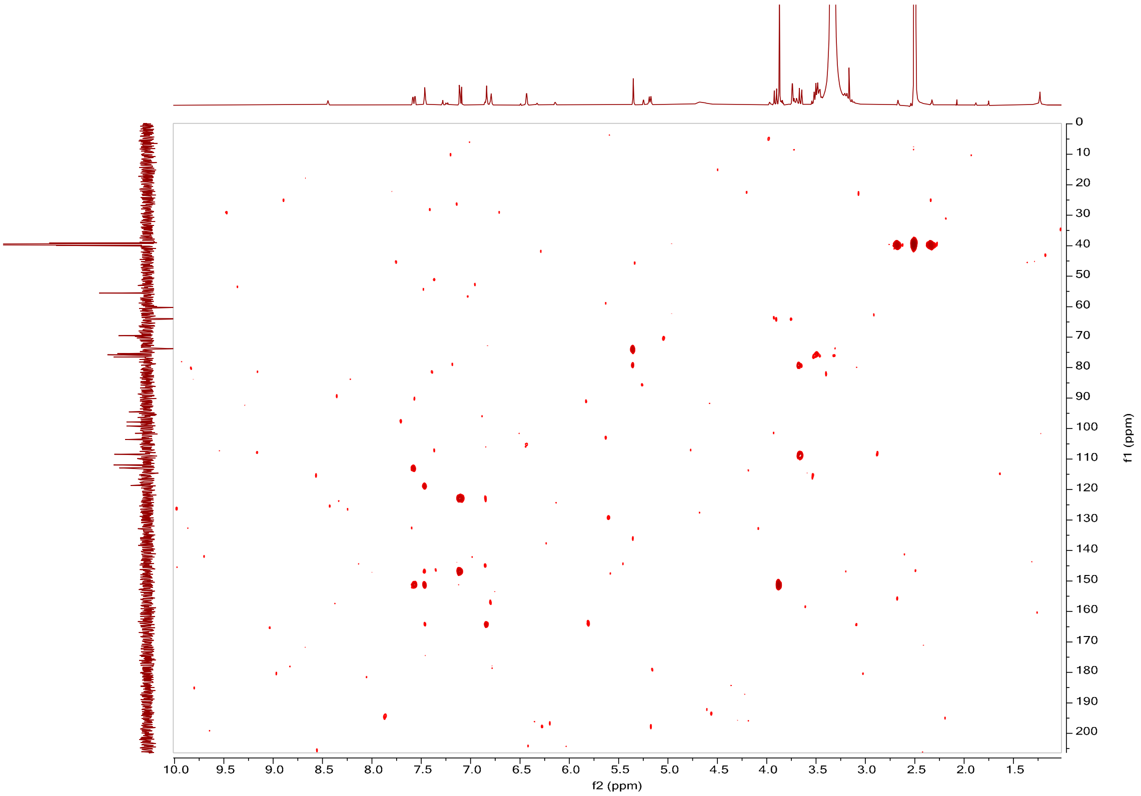

Figure S21. ^{1}H-^{13}C HMBC spectrum of compound **22**.

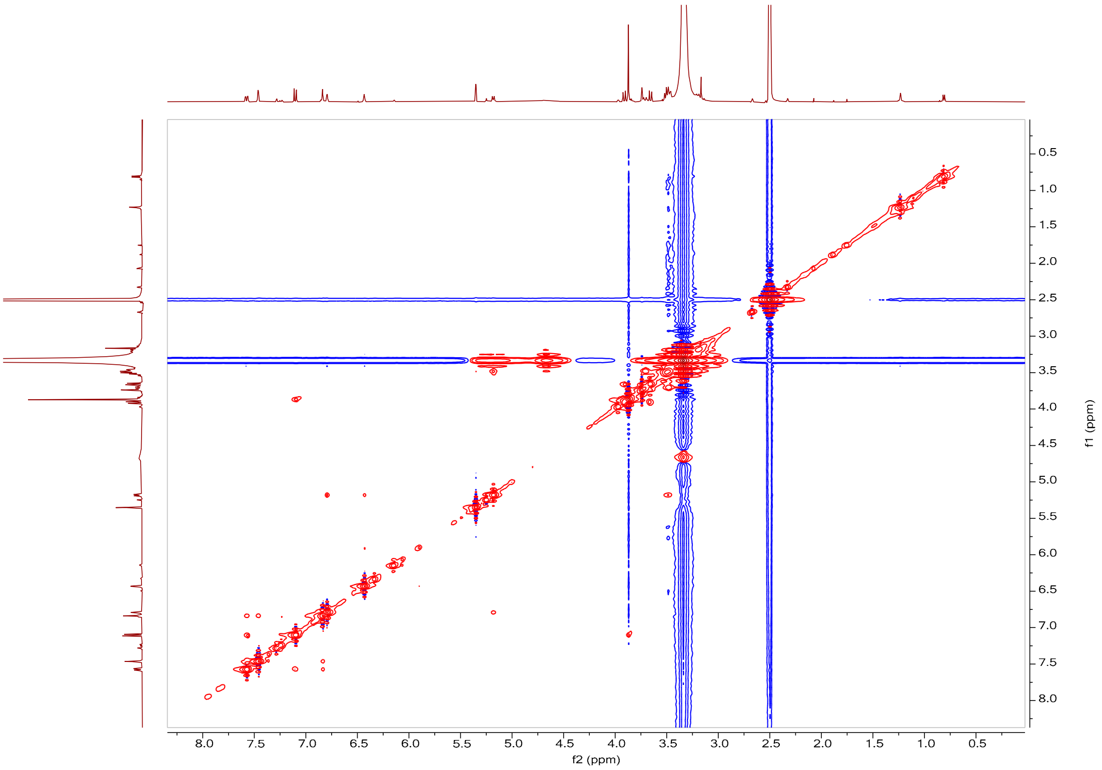

Figure S22. ^{1}H-^{1}H NOESY spectrum of compound **22**.

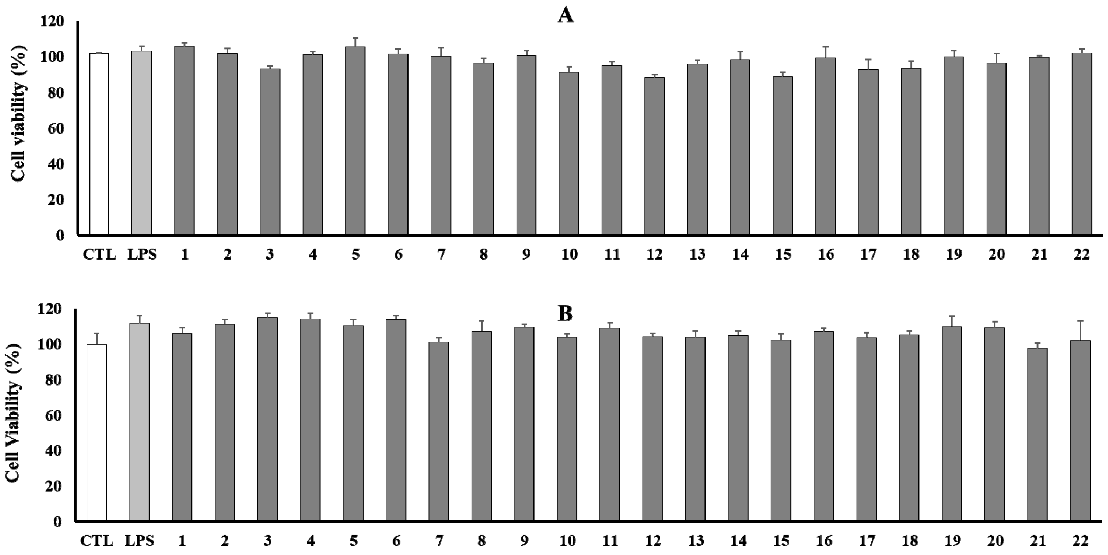

Figure S23. Cell viability of RAW264.7 (**A**) and HT-29 (**B**) by treatment with compounds (**1–22**). Each experiment was performed in triplicates.

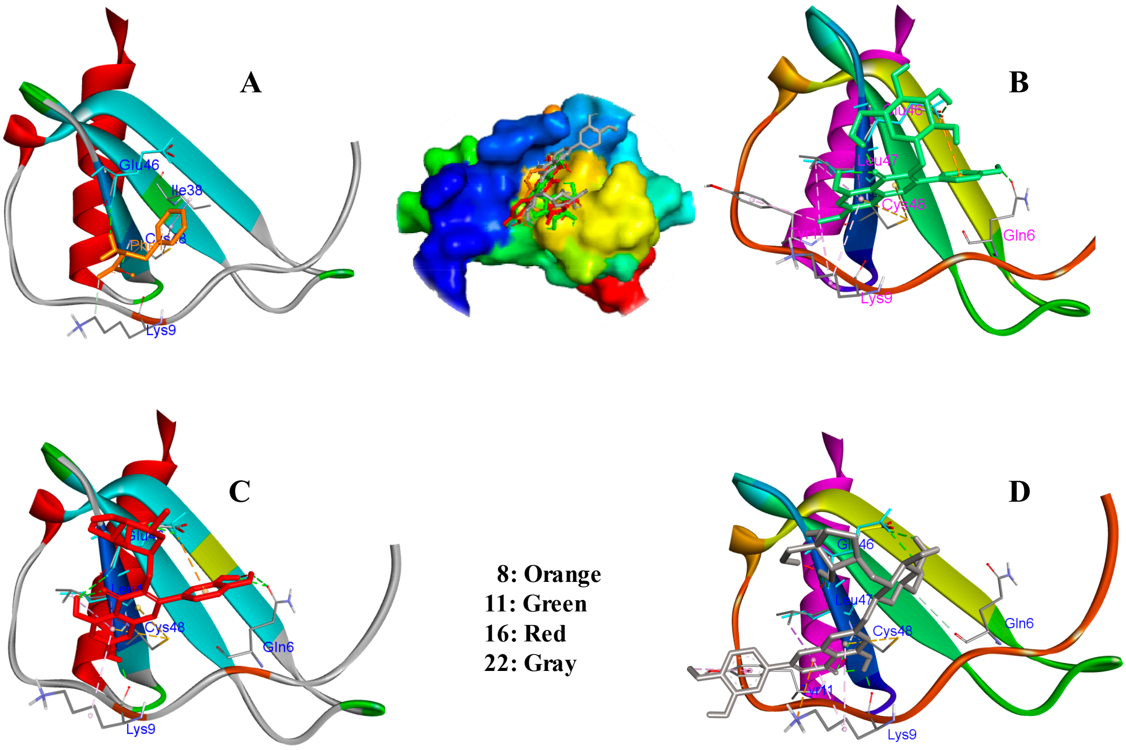

Figure S24. Binding poses and interactions between binding sites of IL-8 receptor with respect to ligands [compounds **8** (**A**), **11** (**B**), **16** (**C**), and **22** (**D**)].
